# Multicomponent Antibiofilm Lipid Nanoparticles as Novel Platform to Ameliorate Resveratrol Properties: Preliminary Outcomes on Fibroblast Proliferation and Migration

**DOI:** 10.3390/ijms24098382

**Published:** 2023-05-06

**Authors:** Giuseppe Angellotti, Giulia Di Prima, Fabio D’Agostino, Emanuela Peri, Maria Rita Tricoli, Elena Belfiore, Mario Allegra, Patrizia Cancemi, Viviana De Caro

**Affiliations:** 1 Department of Surgical, Oncological and Oral Sciences, University of Palermo, 90127 Palermo, Italy; giuseppe.angellotti@unipa.it (G.A.); elena.belfiore@unipa.it (E.B.); 2 Department of Biological, Chemical and Pharmaceutical Sciences and Technologies, University of Palermo, 90123 Palermo, Italy; giulia.diprima@unipa.it (G.D.P.); emanuela.peri@unipa.it (E.P.); mario.allegra@unipa.it (M.A.); patrizia.cancemi@unipa.it (P.C.); 3 Institute for the Study of Anthropogenic Impacts and Sustainability in the Marine Environment, National Research Council (IAS—CNR), Campobello di Mazara, 91021 Trapani, Italy; fabio.dagostino@cnr.it; 4 Department of Health Promotion, Maternal-Childhood, Internal Medicine of Excellence G. D’Alessandro, Section of Microbiology, University of Palermo, 90127 Palermo, Italy; mariarita.tricoli@unipa.it

**Keywords:** resveratrol, glycyrrhetinic acid, menthol, lipid nanoparticles, drug release, kinetic models, wound healing, scratch assay, fibroblasts, antibiofilm

## Abstract

The well-being of skin and mucous membranes is fundamental for the homeostasis of the body and thus it is imperative to treat any lesion quickly and correctly. In this view, polyphenols might assist and enhance a successful wound healing process by reducing the inflammatory cascade and the production of free radicals. However, they suffer from disadvantageous physico–chemical properties, leading to restricted clinical use. In this work, a complex mixture of PEGylated lipid, Glyceryl monoester, 18-β-Glycyrrhetinic Acid and Menthol was designed to entrap Resveratrol (RSV) as the active ingredient and further produce lipid nanoparticles (LNPs) by homogenization followed by high-frequency sonication. The nanosystem was properly characterized in terms of particle size (DLS, SEM), zeta potential, drug loading, antioxidant power (DPPH), release behaviour, cytocompatibility, wound healing and antibiofilm properties. The optimized lipid mixture was homogeneous, melted at 57–61 °C and encapsulated amorphous RSV (4.56 ± 0.04% w/w). The RSV-loaded LNPs were almost monodispersed (PDI: 0.267 ± 0.010), with nanometric size (162.86 ± 3.12 nm), scavenger properties and suitable DR% and LE% values (96.82 ± 1.34% and 95.17 ± 0.25%, respectively). The release studies were performed to simulate the wound conditions: 1-octanol to mimic the lipophilic domains of biological tissues (where the First Order kinetic was observed) and citrate buffer pH 5.5 according to the inflammatory wound exudate (where the Korsmeyer–Peppas kinetic was followed). The biological and microbiological evaluations highlighted fibroblast proliferation and migration effects as well as antibiofilm properties at extremely low doses (LNPs: 22 μg/mL, corresponding to RSV 5 µM). Thus, the proposed multicomponent LNPs could represent a valuable RSV delivery platform for wound healing purposes.

## 1. Introduction

The well-being of skin and mucous membranes is fundamental for the homeostasis of the body as they represent natural barriers against microbial infections while also providing fluid loss control and organ integrity. For this reason, it is imperative to treat any lesion quickly and correctly. After injuries, a complex chain of phases, such as inflammation, proliferation and remodelling, fellow each other into final tissue restoration. During these phases, several processes are involved, such as the release of pro-inflammatory cytokines and chemokines, infiltration of monocytes-macrophages, production of radical oxygen species (ROS), angiogenesis and re-epithelialization [1,2]. However, when one of these phases lasts abnormally (e.g., inflammation), homeostasis is not achieved and consequently the tissue does not repair properly [3]. Nowadays the interest of the scientific community is greatly focused on naturally occurring molecules able to control the inflammatory process by acting as adjuvants to the normal regeneration steps [4]. Several natural anti-inflammatory compounds have been reported, but polyphenols are actually the most interesting class of substances. They are chemically characterized by one or more phenolic group(s), due to which they possess a wide range of therapeutic activities. This is why numerous plants produce polyphenols as a protective response to various stimuli [5]. Among the wide variety of polyphenolic compounds, Resveratrol (RSV) is undoubtedly one of the most studied thanks to its interesting and beneficial properties for human health. RSV is a stilbene found in several dietary sources, such as grapes, wine, peanuts, cocoa, blueberries, bilberries and cranberries [6]. It is commonly produced by plants in response to microbiological infections as it acts as an antimicrobial agent and thereby it is considered a phytoalexin [7,8]. Additionally, numerous researchers have demonstrated its ability to bind different biomolecules, triggering a wide range of effects such as antioxidant [9,10], anti-inflammatory [11], cardio-protective [12] and antitumoral [13,14]. Furthermore, RSV also shows promising wound healing properties. Several studies have demonstrated its ability to induce the proliferation of macrophages, fibroblasts and keratinocytes. Although its mechanism of action is still controversial, it probably acts as an adjuvant by reducing ROS, blocking the inflammatory cascade and maintaining the wound aseptic, instead of directly acting in the regenerative process [15]. Nevertheless, the clinical use of RSV is currently restricted due to its disadvantageous physico–chemical properties, poor water solubility and extensive hepatic metabolism after oral administration [16], leading to handling difficulties and low bioavailability [17,18]. To overcome these drawbacks, RSV encapsulation into carefully designed innovative nanosystems could represent a smart strategy. Nanotechnologies could lead to innovative therapeutic systems able to protect RSV from degradation, promote a controlled and targeted release and enhance its solubility and absorption, thereby increasing bioavailability and, at least, effectiveness [19,20]. Among the variety and complexity of innovative drug delivery nanosystems, lipid-based platforms might be the most appropriate for RSV delivery due to their chemical compatibility with the chosen active. The literature fully reports on the use of solid lipid nanoparticles (SLNs) and nanostructured lipid carriers (NLCs) as first- and second-generation solid lipid nanoparticles, respectively, for several purposes of application [21]. The transition to the second-generation of lipid nanoparticles led to overcoming the main SLNs drawback: the use of solid lipids alone is often inefficient over time due to lipid polymorphism phenomena that involve drug loss during storage. Thereby, NLCs possess enhanced drug loading efficiency and physical stability due to their composition consisting of both liquid and solid lipids at room temperature, leading to amorphous structures [22,23]. Multicomponent lipid nanoparticles (mLNPs) might be considered as a further step in lipid-based nanoparticle innovation. They perfectly merge the previously mentioned positive NLC features with the employment of functional lipophilic components. In particular, considering the high susceptibility of the wound micro-environment to microbial infections, the triterpene Glycyrrhetinic Acid (GA) and the monoterpenoid Menthol (M) could be valid additional hydrophobic functional components to be used in the design of innovative lipid nanoparticles for wound healing purposes, due to their promising antibacterial properties against *Staphilococcus aureus* [24,25]. Furthermore, as it is known that enhancement in drug absorption can be achieved by physical or chemical methods [26], Menthol could be useful for promoting RSV entry into the target tissue as it also possesses penetration enhancer activity [27].

In light of these considerations, the aim of this work was to design a novel delivery platform able to efficiently entrap RSV in a stable form and release it in the tissue microenvironment, where it should exert its beneficial effects. In particular, the formulation of novel multicomponent LNPs constituted by lipophilic excipients, some of which themselves have antimicrobial properties and are able to encapsulate RSV in amorphous form, could represent a winning approach to increase RSV in situ concentration and thus its efficacy as an antioxidant, antimicrobial and regenerative agent. Indeed, when encapsulated in LNPs, RSV should be able to permeate cells and tissues, thus overcoming its low bioavailability caused by the unfavourable physico–chemical characteristics.

The designed platform could generally be useful for several purposes, particularly for wound healing. The goodness of the proposed platform was supported by in vitro release studies in both hydrophilic and hydrophobic environments. The so-designed studies served a dual purpose: first, to validate the proof of concept of LNPs usefulness as RSV delivery platforms for a wide range of applications; secondly, to investigate the LNPs behaviour in simulated wound conditions as the hydrophobic environment mimics the lipophilic domains of tissues, while the acidic aqueous one represents the inflammatory wound exudate. By applying several kinetic mathematical models to release curves, it was also possible to identify the drug discharge mechanism from LNPs. Finally, by in vitro studies against fibroblast (aimed at verifying both the proliferative and pro-migration effects) as well as by microbiological investigations against *S. aureus* (aimed at evaluating the antibiofilm activity), the effectiveness of the proposed RSV-loaded LNPs was demonstrated, thus outlining a new strategy to promote wound healing.

## 2. Results and Discussion

### 2.1. Design, Optimization and Characterization of the Lipid Mixture

Despite the well-known pharmacological potentiality of RSV (e.g., antitumoral, immunomodulatory, cardioprotective, anti-inflammatory, antioxidant, antiviral, etc.) as well as its great abundance in nature [28,29,30,31], its clinical use is significantly restricted due to unfavourable physico–chemical properties, instability, poor water solubility and low bioavailability after oral administration [17,32]. A winning strategy to overcome all the mentioned limitations could be embedding RSV into a valuable delivery platform able to improve its bioavailability, control the release profile and protect it from degradation. Among the variety of innovative delivery strategies, LNPs could be particularly effective due to their chemical compatibility with RSV. Moreover, multicomponent LNPs, the novel generation of solid lipid nanoparticles, are composed of at least two lipids (one solid and one liquid at room temperature), leading to amorphous structures, but they also contain other functional lipophilic excipients chosen to confer some desired and specific properties [33]. The first crucial step for the development of LNPs consists of the optimization of the lipid mixture. Here, three chemically different liquid lipids (IP, CCT and LB) were considered, to identify the best “solvent” for RSV. Unfortunately, IP was quickly discarded because it resulted as being completely unable to dissolve RSV, while CCT (also chosen due to its antimicrobial activities [34]) dissolved an insufficient amount of RSV and was thus also discharged. Finally, LB was selected as the liquid lipid to develop RSV-loaded LNPs as it was able to rapidly and completely dissolve RSV in a desirable amount. This is probably due to its chemical nature: LB is a mixture of capric and caprylic glycerides esterified with polyethylene glycol chains (PEG-8), and it is consequently amphiphilic. Although RSV is a lipophilic compound (LogP 3.1. Source: PubChem https://pubchem.ncbi.nlm.nih.gov/compound/Resveratrol, accessed on 1 February 2023), its hydroxyl groups probably determine the greater affinity towards amphiphilic lipids. Following the identification of the liquid lipid, the second step was the screening of the solid one, which had to be miscible with LB. Again, three chemically different solid lipids (ST, CP and GMS) were considered and mixed at three different weight ratios with LB. ST was unable to mix LB at any ratios, while CP resulted as miscible only when melted but not after solidification, and thus they were both discharged. GMS resulted as being completely miscible (both melted and solidified at any tested ratio) with LB, probably due to the presence of free hydroxylic groups. Additionally, Glycyrrhetinic acid (GA) was also selected as a functional component. GA is a naturally occurring active substance derived from the root of Glycyrrhiza glabra, which possesses a wide variety of beneficial properties (e.g., antiviral, antifungal, antiprotozoal, antibacterial, eupeptic, emollient, antiphlogistic, cytoprotective, etc.) that might act synergistically with RSV [35,36] With the aim of producing a homogeneous lipid mixture, characterized by a good texture and ability to entrap RSV in amorphous form, several lipid mixtures (MIXs) were evaluated, keeping the GA weight percentage fixed (3% *w*/*w*) and varying LB, GMS and RSV ratios (see Section 3). Each MIX was visually evaluated to verify the absence of any crystals and the uniformity both as melted and after solidification. Specifically, MIX-A showed good RSV solubilization and homogeneity when melted. However, an evident phase separation occurred after solidification, probably due to the GMS excess (69% *w*/*w*; LB:GMS ratio = 25:75). MIX-B appeared clear and uniform, both when melted and after cooling. However, the high LB content (>35% *w*/*w*) made the mixture too soft, waxy and difficult to handle. In MIX-C the amount of RSV was increased up to 10% (*w*/*w*), while LB and GMS were reduced. This led to incomplete RSV dissolution. In MIX-D, RSV was fixed at 10% (*w*/*w*) and LB content up to 40%, producing unsatisfactory results similar to MIX-B. In light of the obtained results, it seems clear that to increase RSV content LB is crucial; conversely, an excess of it leads to waxy undesirable mixtures. Additionally, the appropriate ratio of solid:liquid lipids must be chosen to avoid phase separation. Accordingly, MIX-E possessed the desirable characteristics, resulting in a homogeneous appearance, both when melted and after cooling; it was white, smooth and easily workable. To confer further properties to the LNPs, Menthol (M) was also added as a functional component (2% *w*/*w*), obtaining MIX-F. M is a monoterpene which represents the main constituents of Mentha canadensis essential oil. It possesses several properties, such as being analgesic, antifungal, antibacterial, antiviral and anti-inflammatory [37]. Furthermore, it has been proved as an effective RSV permeation/penetration enhancer, able to promote the interactions between RSV and the biological membranes/tissues [38]. However, MIX-F maintained the positive characteristics already observed for MIX-E and was thus chosen as the final lipid composition for LNPs preparation.

Afterwards, the melting point of the chosen MIX, RSV physical state and unaltered RSV amount in the MIX were evaluated as crucial parameters to consider MIX-F suitable for preparing LNPs. The melting temperature range of the lipid mixture could give interesting information regarding the storage conditions required for LNPs. As can be seen in Table 1, MIX-F melted in the temperature range of 57–61 °C.

**Figure 1:**
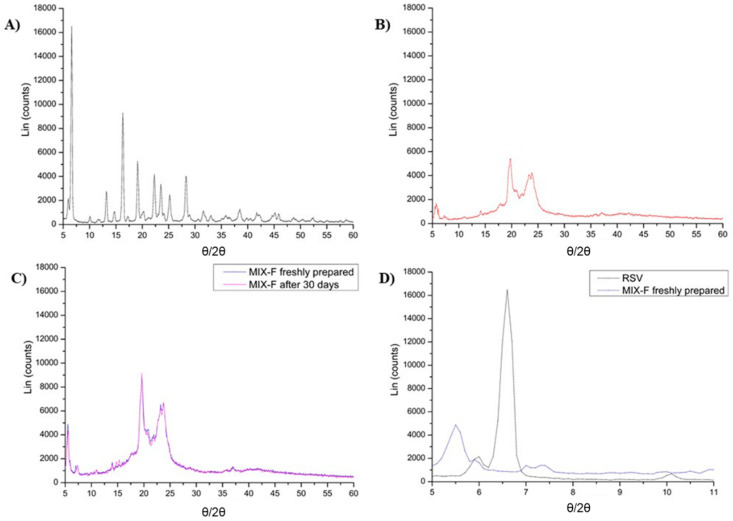
X-ray diffractograms of (**A**) pure RSV (black line); (**B**) MIX-BL (red line); (**C**) MIX-F immediately after preparation (blue line) and after 30 days storage (purple line); (**D**) Magnification of pure RSV and fresh MIX-F diffractograms to better highlight the most representative RSV peak at 6.6 θ.

As can be seen, the melting temperature range of MIX-F is similar to the melting temperature range of the main component GMS (60% *w*/*w*). Furthermore, it should be noted that MIX-F melts at almost 30 degrees above the room temperature; thus it might be considered stable and able to produce solid LNPs at room temperature, which would not require refrigerated storage conditions. The melting temperature of MIX-F led to a further relevant consideration: it is significantly lower than GA and RSV melting points (>200 °C), thus suggesting the complete amorphization of the lipid mixture in general and of the chosen active in particular. The encapsulation of drugs in their amorphous form is always desirable when designing an efficient delivery platform as this could contribute to increasing their solubility and bioavailability. To support this hypothesis, XRD analyses were performed. The diffractogram of pure RSV (Figure 1A) highlighted the characteristic peaks of the crystalline form. In contrast, MIX-BL and MIX-F diffractograms were superimposable **(**Figure 1B,C), suggesting both the obtainment of an amorphous lipid mixture as well as RSV complete amorphization, as confirmed by the absence of its most intense crystalline peak at 6.6 θ (see diffractogram magnification in Figure 1D). Furthermore, it is noticeable that no differences were highlighted after 30 days of storage at 4 °C, confirming the ability of MIX-F in preventing RSV re-crystallization (Figure 1C).

Finally, RSV content in MIX-F was also considered because high temperatures are required to prepare the lipid mixture, and the thermal instability of RSV is well-known. Additionally, by operating the quantitative determination on several randomly selected spots of the same batch of MIX-F, it is possible to evaluate sample homogeneity, while by comparing the results obtained from different batches the reproducibility of the preparation method can be assessed. As a result, the amount of RSV contained in MIX-F was equal to 4.56 ± 0.04% (*w*/*w*) which, compared to a theoretical value of 5.00% (*w*/*w*), validated RSV stability to the operative conditions. Furthermore, the small standard error value confirmed both sample homogeneity and the method’s reproducibility.

### 2.2. Preparation and Characterization of RSV-Loaded LNPs

#### 2.2.1. Particle Size, Z-Potential and Drug Loading

The LNPs were prepared according to the hot-melt top-down method, as previously reported [39,40]. Briefly, the molten lipid mixture was homogenized with an appropriate volume of the aqueous phase, thus producing a microemulsion which underwent ultrasonic treatment until a nano-emulsion was obtained. By cooling the hot nano-emulsion, LNPs were formed.

The effectiveness of this preparation method is closely related to certain parameters, such as the volume and pH of the aqueous phase, type and amount of the surfactants, amount of lipid mixture and instrumental parameters (e.g., homogenization speed and time and sonication parameters which were used according to previous works) [41]. In this context, citrate buffer pH 5.5 was chosen as the aqueous phase because the acidic environment stabilizes RSV [38]. The aqueous phase volume was fixed (40 mL) to allow complete immersion of the sonicator probe. Then, different amounts of MIX-F (100, 200 and 300 mg) were experienced to assess the effect of the increasing lipid mixture amount on the resulting LNPs. The latter was determined by DLS measurements, as reported in Table 2, in terms of average particle size, PDI and Z-potential. The particle size data were reported as Intensity instead of the most commonly used Z-average value because all the samples showed two distinct populations: one was attributable to the LNPs, while the other was related to Pluronic F-127 micelles (16.26 ± 4.32 nm), as confirmed by also carrying out the analysis on the LNPs-free aqueous phase.

**Figure 2:**
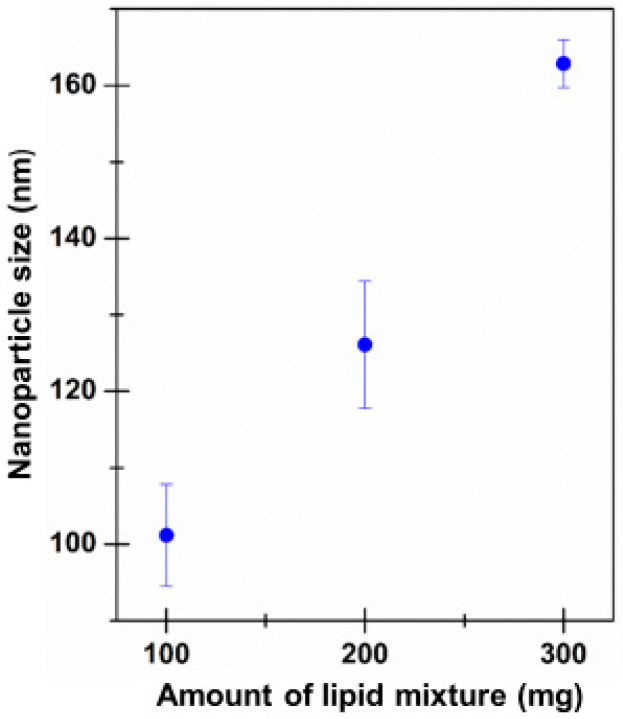
LNP particle size values versus the amount of MIX-F used for LNPs preparation. Mean ± SE (n = 9).

As can be seen, LNP dimensions grew proportionally to the employed amount of MIX-F (Figure 2). However, it should be noted that, in all cases, the particle size values fell within the nanometric range commonly accepted for such drug delivery systems (<500 nm). Furthermore, the PDI values were always acceptable (below 0.4) and the narrow standard error values demonstrated the homogeneity of all the prepared samples [42]. Moreover, it is necessary to underline that the presence of two populations can probably overestimate the PDI. 

The suitability of the preparation method, sample reproducibility and homogeneity, were assessed by RSV quantification in the LNP dispersions. Firstly, the total amount of RSV in the dispersion (sum of the active loaded into the LNPs and the free quota dissolved in the aqueous phase) was determined and reported as DR% (Table 3). The latter always resulted above 95%, thus validating the preparation method, which requires high temperature, but did not determine significant RSV loss due to degradation. The amount of RSV actually encapsulated into the LNPs was then indirectly quantified and expressed as DL% and LE% (Table 3). As a result, the main RSV quota is embedded into the LNPs (LE% > 95%), confirming the great affinity between RSV and the other components of the lipid mixture. These results are satisfactory because a high encapsulation efficiency could allow delivery of significant drug amounts while reducing the preparation costs and the dose to be administered. Moreover, the low standard error values highlighted the reproducibility of the preparation method (data collected on different preparation batches) as well as sample uniformity (data collected on randomly selected portions of the same preparation batch).

Based on the collected results, the LNP-F3 sample was chosen for further studies because it (i) showed optimal DR%, DL% and LE% values; (ii) possessed acceptable particle size and PDI; and (iii) is characterized by the highest amount of MIX-F and consequently RSV, thus reducing the cost and time of preparation as well as the required dose.

**Figure 3:**
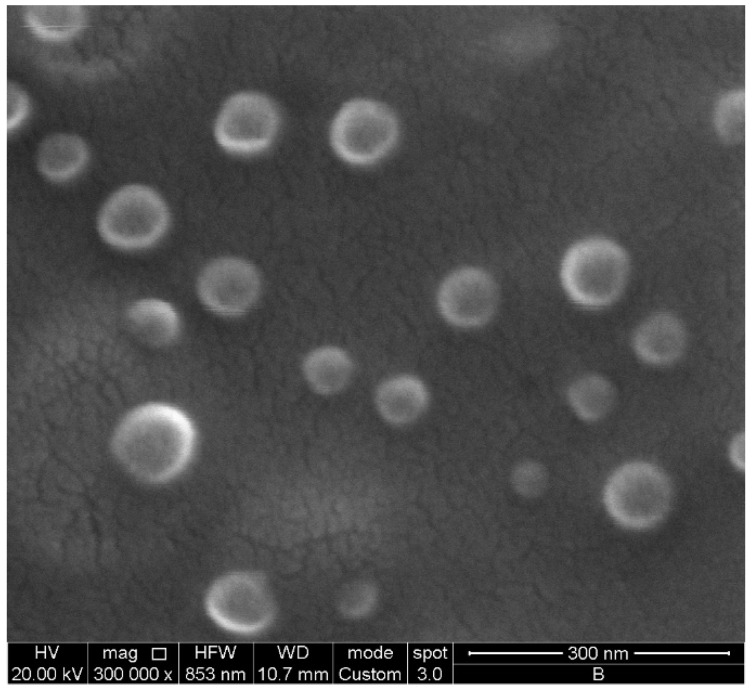
LNP-F3 surface morphology evaluated by SEM analysis; scale bar: 300 nm.

To evaluate the morphology of the chosen LNPs, SEM analyses were further carried out. As a result, spherical, homogeneous and nanometric particles were observed (Figure 3). Moreover, the diameter of the LNPs resulted slightly smaller than that resulting from DLS measurements (≈100 nm vs. ≈160 nm, respectively).

#### 2.2.2. Evaluation of the Scavenger Properties by DPPH Assay

**Figure 4:**
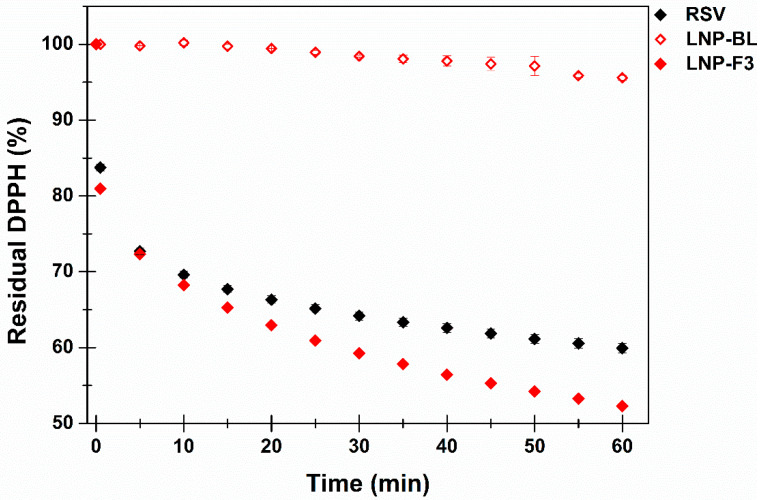
Residual DPPH (%) as a function of incubation time when evaluating the LNP-BL (red—open symbol), LNP-F3 (red—solid symbol) and RSV solution (black—solid symbol). Means ± SE (n = 3).

RSV can indirectly act as an adjuvant of the wound healing process by reducing ROS, blocking the inflammatory cascade and maintaining the wound aseptic. Particularly, ROS reduction is directly related to RSV antioxidant power, and thus the evaluation of the LNPs scavenging properties could predict their functional anti-radical effect. The DPPH assay, a simple, well-known and widely used technique, was chosen to compare the antioxidant behaviour of the RSV solution as well as RSV-free LNPs and RSV-loaded nanoparticles (Figure 4) [43].

As a result, LNP-BL showed a minimal scavenger effect, probably due to the presence of GA in the lipid mixture. In contrast, RSV-loaded nanoparticles (LNP-F3) exhibited a time-dependant significant anti-radical behaviour. The latter resulted slightly higher than the one observed for the same concentration of free-RSV, thus suggesting the additive effect of RSV and GA to the scavenger properties of the final nanosystem.

#### 2.2.3. Long-Term Storage Studies: Freeze Drying and Choice of the Cryoprotectant

A valuable strategy to cut costs could be drying the dispersion producing solid formulations, which are more stable over time and can be more easily stored and transported. Indeed, the presence of water could determine many disadvantages, such as difficulty of administration, physical and microbiological instability, chemical degradation and expensive storage conditions. In addition, a dry pharmaceutical product is preferable in the regenerative medicine field, as it is easier for it to be sterilized, dosed and applied [44,45]. The most commonly used drying technique is freeze drying, which is extremely useful for thermolabile substances, such as polyphenols, and allows one to obtain dry, highly porous, hygroscopic and easily re-dispersible powders [46]. However, during the freezing phase the formation of ice needles cloud break or deform the LNPs. To overcome this issue, cryoprotectants can be an easy solution as they can position themselves between LNPs and water molecules, acting as protective pads. In addition, the adsorption of the cryoprotectant on the LNPs surface produces a steric effect which can prevent or minimize aggregation during the freeze-drying process. As expected, the here-proposed RSV-loaded LNPs are not able to be dried by themselves (Table 4, LNP-NC sample), and consequently four different cryoprotectants (sucrose, trehalose, mannitol and sorbitol) were evaluated at three weight ratios. The effectiveness of the selected molecules was assessed on LNPs freeze dried dispersions by visual investigation (appearance of the resulting dispersion and time required for the re-dispersion process) and DLS analysis. The analyses gave mutually consistent results. In particular, LNPs subjected to freeze drying in the absence of cryoprotectant were enormously difficult to re-disperse, even by ultrasound or vortexes, resulting in large, suspended particles which tended to precipitate over time. Mannitol insertion did not significantly modify the latter trend, while trehalose, sorbitol and sucrose gave better results. Particularly, trehalose and sorbitol produced a homogeneous and slowly re-dispersible solid which rapidly became cloudy. In contrast, sucrose allowed a fast re-dispersion (almost immediate) of freeze-dried LNP-F3 and also had an appearance comparable to the same fresh sample. These observations agreed with the DLS results reported in Table 4. 

Based on the DLS data, LNP-Su (B) was chosen for further studies as it showed satisfying efficacy by 1:8 (*w*/*w*) ratio of LNPs/cryoprotectant, avoiding an excess of sugar. It should be noted that, despite the fact that the resulting LNPs cryoprotected by sucrose were larger than the freshly prepared ones (340.35 vs. 162.86 nm), their PDI value remained almost unchanged. Moreover, the enlargement of the freeze-dried LNPs could be related to the Z-potential variation that was also observed during the DLS analysis. Indeed, the negative charge of the freshly prepared dispersion was initially maintained in the presence of sucrose 1:8 (*w*/*w*) prior to freeze drying (−21.40 ± 7.33 vs. −23.70 ± 9.27 mV) but dramatically decreased after re-dispersion of the freeze-dried sample (−1.50 ± 5.80 mV). This could be attributable to sucrose adsorption onto LNPs surfaces, thus modifying their charge and leading to particles aggregation. However, these results were still considered satisfactory (particle size < 500 nm), also because no significant changes in terms of DR%, DL% and LE% were observed when compared with the freshly prepared sample (Table 5).

Additionally, the presence of sucrose in the dry formulation could determine some further advantages. On one hand, recent studies confirmed that the in vivo use of sucrose-loaded xyloglucan hydrogels and films might have a beneficial healing effect against deep wounds [47]. On the other hand, sucrose will enhance the osmotic pressure in the application site, thus promoting the adsorption of the exudate and minimizing the oedema typically present in several inflammatory stages [48].

### 2.3. RSV Release Studies and Kinetic Evaluations

Generally, conventional dosage forms are subjected to standardized in vitro protocols, as described by the Pharmacopoeia, while alternative methods have been proposed to study the release mechanism from unconventional delivery systems such as nanoparticles [49,50]. To correctly design a drug release study, it is mandatory to define experimental conditions (e.g., sink conditions, suitable solvents, etc.) that are fundamental to maintaining the driving force guiding the release phenomenon. As release tests often give method-dependent results, different experimental approaches should be considered when validating a novel delivery platform. As a consequence, the LNPs were tested in both hydrophobic and hydrophilic environments and, in both cases, data were curve fitted to several mathematical models to determine the main mechanism governing RSV release. The release assays were carried out on the following samples: the best freshly prepared LNP dispersion (LNP-F3, indicated as LNP), the LNP-F3 freshly prepared dispersion containing sucrose (1:8 *w*/*w*) (indicated as LNP-Su) and the sample subjected to freeze drying and then re-dispersion (indicated as LNP-Su-R).

#### 2.3.1. RSV Release Studies to Hydrophobic Acceptor Fluids

Hydrophobic acceptor fluids were chosen to mimic the interaction of the RSV-loaded LNPs with lipophilic target tissues (e.g., epithelial membranes) as well as to assess their aptitude to accumulate into those. As the aim of these experiments was to evaluate the LNPs as a whole, the modified Transwell system was chosen as an open bicompartmental model [28]. In particular, a Transwell insert with a cut off of 0.45 µm was chosen to allow the passage of the whole LNPs instead of RSV by itself from the aqueous donor fluid to the lipophilic acceptor one. As acceptor fluids isopropyl myristate and 1-octanol were selected as they are well-known to mimic biological tissues [40,51,52]. Unfortunately, isopropyl myristate caused interferences with RSV detection and was therefore discarded. Therefore, the LNP release trend was evaluated in 1-octanol by plotting the RSV dose fraction detected in the acceptor compartment as a function of time (the graphical presentation of the data is reported below, together with the kinetic evaluations and the best curve fitting).

Generally, it should be noted that, despite the high affinity of the LNPs for the acceptor fluid, the release trend of all samples was not completed after 48 h. However, the experiments were stopped because the plateau was reached. Such differences were observed in the RSV release rate among the tested LNP samples, probably directly related to the different characteristics of the samples. In particular, the re-dispersed sample (LNP-Su-R) displayed the fastest release rate. This could be related to the bigger dimension of the freeze-dried LNPs, leading to a greater influence of the gravity, thus resulting in the enhanced approaching of the LNPs to the bottom acceptor compartment. By adding sucrose to the freshly prepared dispersion (LNP-Su), the slowest release rate was observed. This phenomenon might be related to the presence of a large hydrophilic shell within the LNPs, which hinders their passage to 1-octanol. 

#### 2.3.2. RSV Release Studies to Hydrophilic Acceptor Fluids

Hydrophilic acceptor fluids were chosen to simulate the aqueous microenvironment of the wound exudate. Two different hydrophilic acceptor liquids were employed: citrate buffer pH 5.5 and the same medium supplemented with 0.1% (*w*/*v*) of β-CD, chosen as a well-known solubilizing agent for RSV. The pH of 5.5 allows the maintenance of RSV stability over time and mimics the acidic environment related to the inflammatory processes during the wound early stage [53]. The addition of β-CD can mimic the presence of elements (e.g., proteins) in the wound exudate that are able to interact and complex RSV, thereby increasing its solubility and rate of solubilization and thus apparently accelerate the release process [54]. To perform these studies, the dialysis method was chosen to prevent the diffusion of the whole LNPs while allowing only free RSV discharge.

The results were expressed as RSV dose fraction released from the LNPs as a function of time (the graphical presentation of the data is reported below, together with the kinetic evaluations and the best curve fitting). 

It was observed that the RSV release rate is greatly influenced by the acceptor medium. In particular, in citrate buffer pH 5.5, the RSV release rate from LNPs appeared very slow (complete discharge after 24 days), probably due to the low affinity of RSV for the aqueous environment. In agreement with this, the presence of β-CD significantly increased the release rate (complete discharge after 48 h) due to the high affinity of RSV towards β-CD, which consequently acts as a sequestrant agent. Furthermore, it is worth noting that, in both cases, the LNP and LNP-Su samples displayed superimposable results. This could be related to their similarity in terms of particle size and Z-potential, which are not affected by the presence of sucrose. Furthermore, sucrose is free to diffuse in the hydrophilic acceptor medium, thus diluting and exerting no influence. In contrast, the LNP-Su-R sample showed the slowest release rate when the acceptor fluid was citrate buffer alone. This result is probably due to the larger particle size reducing the total surface area through which RSV could be released. Furthermore, given the slow release of RSV in this medium (days), it is possible that the particles further agglomerate in this long period of time, according to the Z-potential variation, resulting in a very slow drug discharge. However, the latter phenomenon is not highlighted in the presence of β-CD because the greater rate of RSV release (hours) may not allow aggregation phenomena or not make them preponderant.

#### 2.3.3. Kinetic Evaluations

The release data were curve-fitted to several kinetic models to identify the main mechanism governing RSV release. To acquire more complete information, further parameters, such as T_lag_ (T_lag_ > 0 indicates an actual lag time; T_lag_ < 0 indicates a burst effect) and F_MAX_, were considered when needed. The experimentally determined points were curve fitted until different end points were reached: generally, the studies performed to citrate buffer pH 5.5 supplemented with β-CD and 1-octanol were both elaborated until the plateau was reached (26–28 h) and until the end of the experiments (48 h), while those performed to citrate buffer pH 5.5 were considered until the last time point at 24 days. The kinetic models considered are reported in Table 6.

**Figure 5:**
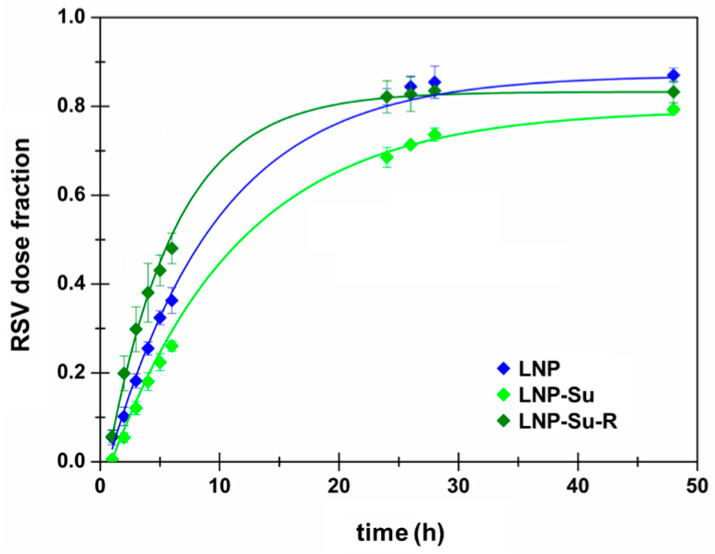
RSV release profile from LNP dispersions to 1-octanol, reported as dose fraction as a function of time until 48 h. Data were curve-fitted to the First Order model also considering the T_lag_ and F_MAX_ parameters. Mean ± SE (n = 3).

As expected, the two different environments of the experiments gave dissimilar results in terms of adherence to the kinetic models. In particular, RSV release from LNPs to 1-octanol is well described by the First Order model when considering both T_lag_ and F_max_ parameters. The results are reported in Figure 5 and Table 7.

It is likely that the whole LNPs diffuse from the donor aqueous fluid to 1-octanol, instead of an actual RSV release and free RSV partition. As a consequence, a diffusion process might occur, and this certainly depends on the LNP concentration in the donor compartment. Additionally, the obtained *lag time* could be related to the time required for LNPs to move from the aqueous donor medium to the lipophilic acceptor ones. Finally, it should be highlighted that in all cases the presence of sucrose as well as the freeze-drying process can only slightly affect the release rate, while they did not modify the release mechanism.

**Figure 6:**
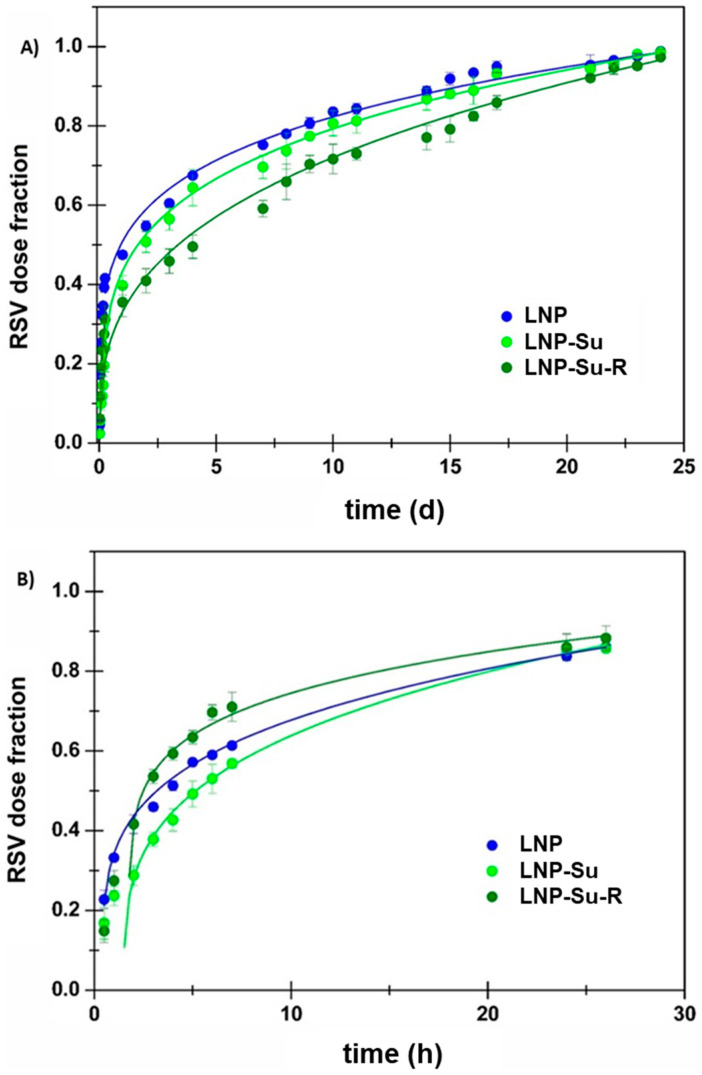
RSV dose fraction as a function of time released from LNPs to (**A**) citrate buffer pH 5.5, until 24 days, and (**B**) citrate buffer supplemented with 0.1% (*w*/*v*) of β-CD, until 48 h. Data were curve-fitted to the Korsmeyer–Peppas model while also considering the T_lag_ parameter. Mean ± SE (n = 3).

On the other hand, the RSV release behaviour to the hydrophilic environment was well described by the Korsmeyer–Peppas model when the lag time parameter was considered (Figure 6, Table 8 and Table 9). Despite the fact that the presence of β-CD increased the release rate, it did not affect the release mechanism, confirming that this behaviour is not method-dependent. 

It is interesting to note that the experimental “*n*” values were always below 0.5 (0.2 < *n* < 0.4). These results are anomalous for this kinetic model. However, similar behaviours have been reported in the recent literature to describe the discharge phenomenon from nanoparticles. In particular, it was observed that when *n* < 0.5, a non-Fickian release mechanism occurs, and this is characteristic for those drug delivery systems which have a barrier (e.g., surface coating) that limits solvent entry and thus drug dissolution rate [61]. Of course, the lipophilic nature of the LNPs can acts as a barrier against water, thereby hindering RSV release, as also confirmed by the presence of a lag time.

### 2.4. Biological Evaluations

**Figure 7:**
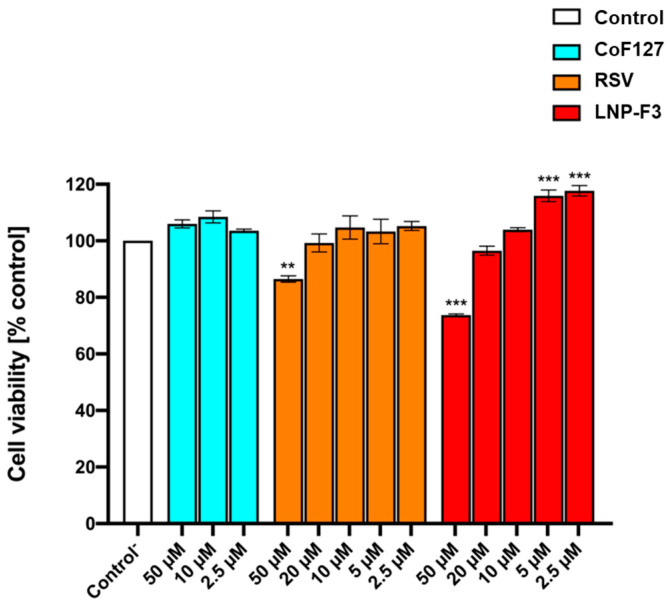
Cell viability assay on BALB/3-T3 cells. Cells were treated for 24 h in the absence (control) or presence of either citrate buffer pH 5.5 *plus* Pluronic F-127 (Co F127), RSV or LNP-F3 dispersions at different concentrations (from 2.5 to 50 µM). Values are means ± SD of three separate experiments conducted in triplicate (n = 9). With respect to control, ** *p* < 0.01; *** *p* < 0.001 (ANOVA associated with Tukey’s test). The Co F127 sample was evaluated at the same dilution as the LNP-F3 samples needed to achieve 2.5, 10 and 50 μM of RSV.

To assess the safety of the proposed delivery platform, the cytotoxicity assay against murine fibroblasts (BALB/3-T3 cells) was evaluated. Cells were treated with increasing concentrations of either RSV or freshly prepared LNP-F3 dispersions, equal in terms of RSV amount (from 2.5 to 50 µM). Additionally, three concentrations of the LNPs-free and RSV-free aqueous phase used to prepare the nanoparticles, i.e., citrate buffer pH 5.5 *plus* Pluronic F-127, were tested to determine any cytotoxic effects of the surfactant (see Section 3). Data are reported in Figure 7 in terms of cell viability%, referring to untreated control cells. 

The obtained results showed that the chosen surfactant was completely cytocompatible at the tested concentrations, in accordance with the literature data [62]. Free RSV also resulted as cytocompatible in the range of 2.5–20 µM, while it induced a decrease in cell viability at the highest tested concentration (50 μM). This evidence is in line with the literature and, specifically, with the widely reported double-sided effects of RSV on cell proliferation. Polyphenols are, indeed, well known to be safe and, generally, to possess wound healing and proliferative properties at low concentrations, while they seem to exert anti-proliferative and cytotoxic effects at high doses, so much so that they are proposed as adjuvants in anticancer treatment [63,64,65]. Predictably, the RSV-loaded LNPs magnified the discussed pattern. Indeed, the reported data showed a pronounced proliferative effect at lower concentrations (corresponding to RSV 2.5 and 5 µM) and a decrease in cell viability at the highest tested concentration (50 µM). Clearly, the proposed LNPs are able to enhance RSV actions, thus maximizing its dual behaviour. This is probably due to their ability to interact with biological membranes and cells, thus promoting RSV entry, bioavailability and resulting effects [66].

**Figure 8:**
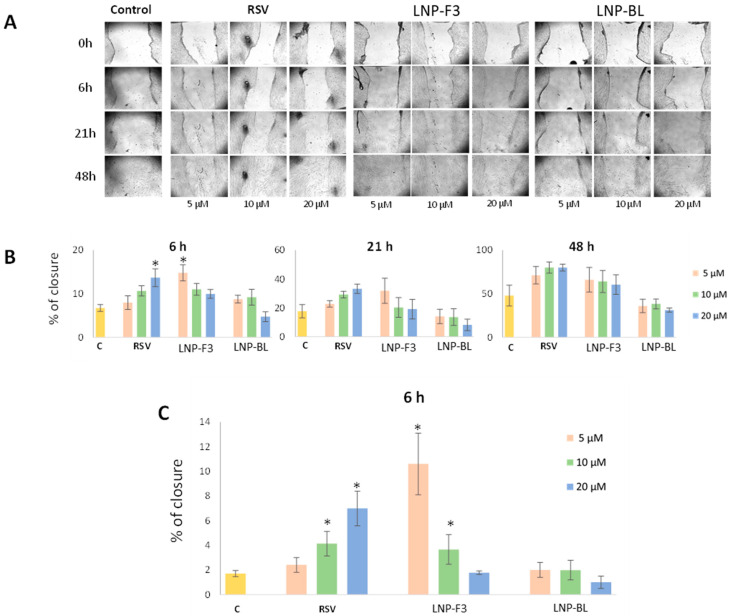
Effect of RSV, LNP-F3 and LNP-BL on cell migration by the wound healing scratch assay. Normal fibroblast IMR-90 cells were treated with 0, 5, 10 and 20 µM of free RSV and corresponding doses of LNP-F3 and LNP-BL. (**A**) Representative images of the wound healing assay over time in the presence of FBS 10% after 0, 6, 21 and 48 h of migration. Magnification, ×40: (**B**) Graph showing the scratch wound closure monitored over time (* *p* < 0.05) referred to (**A**) experiments; (**C**) Graph showing the scratch wound closure monitored after 6 h of treatment by using the FBS-free cell culture medium (* *p* < 0.05). Panels B and C are reported as means ± SD (n = 6).

Further biological studies were conducted as a proof of concept of the potentiality of RSV-loaded LNPs for wound healing purpose. The wound healing properties of LNP-F3 were evaluated in terms of normal fibroblast migration by the scratch assay. Cells were treated with increasing concentration of RSV as well as freshly prepared LNP-F3 dispersion, equal in terms of RSV amount (5, 10 and 20 µM). Additionally, the RSV-free LNPs (LNP-BL) were evaluated to verify any vehicle property. Two different sets of experiments were then carried out by treating cells in the presence (Figure 8A,B) or absence (Figure 8C) of FBS. The FBS variable was studies because the latter stimulates cell proliferation (especially for longer time points), thus specifically impairing the ability of evaluating cell migration. The obtained data fully confirm this statement. Indeed, in the presence of FBS, cell migration was monitored at 6, 21 and 48 h. As can be seen (Figure 8), the best performance was recorded after 6 h of treatment. In detail, the lower LNP-F3 concentration (corresponding to RSV 5 μM) significantly increased the migratory capability of fibroblasts in terms of wound closure. On the contrary, free RSV induced the stimulation of the migratory capability in a dose-dependent manner. The monitoring of wound closure at higher time points (21 and 48 h) did not reveal significant differences compared with untreated cells, although the same trend was maintained. This is probably related to normal cell proliferation, which prevails over cell migration, thus hiding it and leading to misleading results. As a consequence, the same experiments were also performed by using serum-free cell culture medium (Figure 8C). However, in this way, only the shortest time point could be evaluated because, for prolonged incubation times, cells could no longer survive. Again, a clear dose-dependent effect was recorded for free RSV, and an inverse trend was detected for LNP-F3. Both in the presence and absence of FBS, no significant effect was detected following RSV-free nanoparticle treatment. As already suggested by the cytocompatibility assay, the different behaviour of free RSV and LNP-F3 could be explained by different drug bioavailability. The lipid nanoparticles, indeed, could significantly promote RSV entry into cells, thus strongly enhancing its effects, leading to distinct observation of its dual comportment (wound healing at lower doses, antimetastatic at higher doses). Clearly, the LNPs greatly magnify RSV effects, and this could lead to interesting wound healing properties, also when administering extremely low doses (RSV 5 μM corresponds to an LNP concentration equal to 22 μg/mL).

### 2.5. Microbiological Evaluations

**Figure 9:**
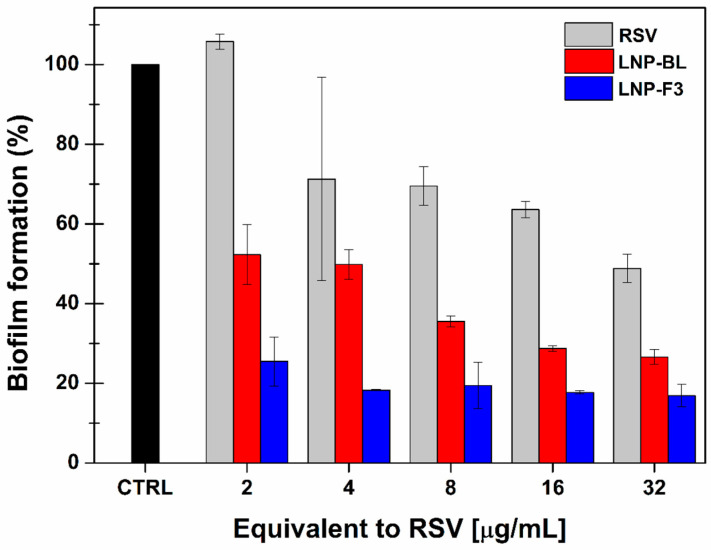
Biofilm formation % by *S. aureus* (ATCC 12973) after a 24 h treatment with free RSV solution (gray), LNP-BL (red) and LNP-F3 (blue) at increasing concentrations (equivalent to RSV 2, 4, 8, 16 and 32 µg/mL). The percentage of biofilm formation refers to the untreated control cells (black), considered as 100%. Mean ± SE (n = 3).

Biofilm infections are a pernicious factor in human health. Biofilms are hostile microbial aggregates where bacteria have gained tolerance to antibiotics and host immune defences. They can infect wounds, even when closed, leading to alteration of the extracellular matrix and impairment of the whole barrier function, thus causing wound recidivism. When the biofilm formation occurs, the use of topical and parenteral antimicrobial agents without wound debridement displayed only a limited impact on decreasing biofilm infection, which remains a major problem in wound care [67]. The literature fully reports on the antibiotic, antifungal and antiviral activity of RSV. Moreover, its antibiofilm dose-dependent effects against several Gram-positive and Gram-negative bacteria have also been highlighted [68,69,70]. As previously observed by in vitro studies, by embedding RSV into the LNPs, it is possible to enhance its therapeutics properties, probably due to improved bioavailability. Additionally, the composition of the prepared multifunctional LNPs should not be overlooked because the chosen functional ingredients (Glycyrrhetinic acid and Menthol) possess antimicrobial activity and could thus act in synergy with the loaded RSV. Figure 9 shows the inhibitory effect on biofilm formation by *S. aureus* when testing free RSV (DMSO solution), RSV-free LNPs (LNP-BL) and the optimized RSV-loaded nanosystem (LNP-F3). As can be seen, RSV displayed a dose-dependent inhibitory effect, according to the literature. Interestingly, the LNP-BL sample showed intrinsic antibiofilm properties which were further enhanced by loading the active substance: the LNP-F3 formulation resulted the most effective one due to the synergy of RSV and its vehicle. Additionally, it should be noted that both free-RSV and LNP-BL exhibited a concentration-dependent trend, while the RSV-loaded LNPs gave almost the same effect as all the tested concentrations. As a consequence, the proposed lipid nanoparticles should be effective at extremely low doses to achieve both the wound healing and antibiofilm actions, as desired in the management of wounds.

## 3. Materials and Methods

### 3.1. Materials

*Trans*-Resveratrol (RSV), 18-β-Glycyrrhetinic Acid (GA), β-Cyclodextrin (β-CD) and Isopropyl Palmitate (IP) were purchased from A.C.E.F spa (Fiorenzuola D’Arda, Piacenza, Italy). Pluronic F-127 was supplied by Sigma Aldrich (Milan, Italy). Glyceryl Monostearate 55–60 (GMS), Sorbitol, Sucrose, Mannitol, Isopropyl Miristate (IM), Stearin (ST) and Caprylic/Capric triglyceride (CCT) were obtained from Farmalabor (Canosa di Puglia, Italy). Labrasol^®^ (LB) and Cetyl Palmitate (CP) were kindly supplied by Gattefossé (Lyon, France). Menthol (M) and 1-Octanol were purchased from Carlo Erba Reagents (Milan, Italy). Trehalose was purchased from Hayashibara Shoij (Okayama, Japan). Trifluoroacetic acid (TFA) was obtained from Merck (Darmstadt, Germany).

The citrate buffer pH 5.5 was prepared by dissolving 2.052 g of sodium citrate dihydrate and 0.636 g of citric acid monohydrate in 1 L of distilled water.

All chemicals and solvents (analytical grade) were purchased from Carlo Erba Reagents (Milan, Italy) and were used without any further purification.

### 3.2. Preparation of RSV-Loaded Lipid Mixtures

#### 3.2.1. Screening of the Liquid Lipid

An amount of 50 mg of RSV was added to 950 mg of each tested liquid excipient and kept under constant stirring (200 rpm) at 40.0 ± 0.5 °C (Heidolph MR3001K Hotplate Stirrer with probe temperature Heidolph EXT3001, Heidolph Instruments, Schwabach, Germany) until RSV complete solubilization occurred or until any visible modification was detectable. Three chemically different liquid lipids (IP, CCT and LB) were tested, each in triplicate (n = 3). The obtained solutions or dispersions were subjected to visual analysis, both during the dissolution assay and after cooling down at room temperature.

#### 3.2.2. Screening of the Solid Lipid

The screening of the solid lipid was based on the previously selected liquid lipid (LB). A carefully weighted amount of LB was placed into a beaker, and then the tested solid lipids (ST, GMS and CP) were added at three different weight ratios as follows: LB:Solid lipid as 40:60, 50:50 and 60:40. The mixtures were heated to 60.0 ± 0.5 °C and maintained under constant stirring (Heidolph MR3001K Hotplate Stirrer with probe temperature Heidolph EXT3001, Heidolph Instruments, Schwabach, Germany) until the solid lipid melted. The obtained lipid mixtures were visually analysed, both during the assay and after cooling down at room temperature. Each experiment was performed in triplicate (n = 3).

#### 3.2.3. Optimization of the Lipid Mixture

Once the main liquid and solid excipients were defined, different lipid mixtures were prepared by mixing LB, GMS, GA, M (when present) and RSV at several weight ratios (Table 10).

Briefly, the appropriate and carefully weighted amount of RSV was dissolved in LB under constant stirring (200 rpm) at 40.0 ± 0.5 °C (Heidolph MR3001K Hotplate Stirrer with probe temperature Heidolph EXT3001, Heidolph Instruments, Schwabach, Germany). Subsequentially, the temperature was increased up to 60.0 ± 0.5 °C and the other excipients were added in the following order: GMS, GA and M (when present). The whole mixture was maintained under constant stirring until complete melting. The melted mixtures were placed into an ice/water/NaCl bath for 5 min, then cooled down at −20 °C for 15 min and stored at 4 °C after solidification. Accordingly, RSV-free blank mixture (MIX-BL) was prepared based on the MIX-F composition. Each mixture was prepared in triplicate (n = 3) and visually investigated in terms of appearance and homogeneity, both when melted and after solidification.

### 3.3. Evaluation of the Melting Temperature Range of the Optimized Lipid Mixture

The melting temperature range was determined by placing randomly selected aliquots of MIX-F into a capillary glass and analysing them by a Büchi Melting Point B-540 instrument (Flawil, Switzerland). Samples were placed into the instrument at room temperature, and then a heating rate of 5 °C/min was applied until complete melting. Similarly, the melting point of each single component were determined. The analyses were performed in triplicate, and results are reported as means ± SE (n = 3).

### 3.4. X-ray Diffraction (XRD) Analysis

The physical state of MIX-F was assessed by an X-ray diffractometer (D-8 Focus, Bruker, Billerica, MA, USA). Diffractograms were obtained from 5° to 60° in ⊖/2⊖ at 2°/min, 40 kV voltage and 30 mA current at room temperature. XRD analyses were performed in triplicate (n = 3) on pure RSV and randomly selected aliquots of MIX-BL and MIX-F immediately after their preparation, as well as after 30 days of sample storage (4 °C, in the dark).

### 3.5. Determination of RSV Amount into the Optimized Lipid Mixture

An amount of 5 mg (analytical balance mod. AE 240, MettlerToledo S.p.A., Milan, Italy) of MIX-F was randomly withdrawn from two different parts of the lipid mixture (radial and central), placed into a volumetric amber flask and dissolved in methanol with the aid of an ultrasound bath (Branson 1200, Branson Ultrasonics, Danbury, CT, USA). The resulting clear solution was subjected to spectrophotometric measurements, as described below. The experiment was performed in duplicate on three different lipid mixtures immediately after their preparation (n = 6). The results are expressed as percentage amount (*w*/*w*) of RSV into the lipid mixture and reported as means ± standard error (SE).

### 3.6. Preparation of RSV-Loaded LNPs

The RSV-loaded and RSV-free LNPs were prepared according to the homogenization followed by the high-frequency sonication method, as previously described [40]. Briefly, the chosen carefully weighted amount of MIX-F or MIX-BL was melted and emulsified with 40 mL of the aqueous phase (citrate buffer pH 5.5 containing 400 mg of Pluronic F-127) pre-heated at 70.0 ± 0.5 °C using a homogenizer (Kinematica Polytron Model PT MR 2100, Kinematica AG, Malters, Switzerland) at 19,000 rpm for 1 min. The resulting hot coarse emulsion was subjected to high-frequency sonication (Sonopuls, Bandelin, mod. HD 2070, Berlin, Germany) at 20 kHz frequency, 88–90% amplitude and in pulse condition (cycles of 0.7 s of activity and 0.3 s of inactivity) for a total of 10 min. This procedure was repeated twice, first at room temperature and subsequently by placing the dispersion into an ice/water/NaCl bath, allowing solidification of the LNPs. The composition of each dispersion is reported in Table 11. Each dispersion was prepared in triplicate (n = 3).

### 3.7. Quantitative Determination of RSV into the LNP Dispersions

#### 3.7.1. Determination of RSV Total Amount and Drug Recovery% (DR%)

An amount of 100 µL of the fresh RSV-loaded LNP dispersions was placed into a 10 mL volumetric amber flask and dissolved with methanol. The resulting clear solution was subjected to UV-Vis analyses, as reported below, to quantify the total amount of RSV into the whole dispersion (RSV loaded into the LNPs + free RSV). Results are reported as Drug recovery % (DR%), calculated as follows:

$$\mathrm{DR}\%=\frac{{\mathrm{RSV}}_{\mathrm{tot}}\;\left(\mathrm{mg}\right)}{{\mathrm{RSV}}_{\mathrm{MIX}-F}\;\left(\mathrm{mg}\right)}\times 100$$

where RSV_tot_ is the amount of active (mg) recovered into the whole LNP dispersion and RSV_MIX-F_ is the amount of RSV employed for the preparation based on the RSV amount into the lipid mixture (MIX-F). The analyses were performed in triplicate on each prepared batch of LNPs (n = 9), and results are reported as means ± SE. 

#### 3.7.2. Drug Loading% (DL%) and Loading Efficacy% (LE%)

The amount of RSV encapsulated into the LNPs was indirectly evaluated, as previously reported [41]. In brief, 1 mL of fresh LNP dispersion was diluted 10-fold with citrate buffer pH 5.5. Then, 0.5 mL of the obtained dispersion was placed into the upper chamber of a centrifuge filter tube equipped with an inert porous membrane (Ultrafree-MC, Millipore, Burlington, MA, USA, cut off 30,000 Da) and centrifuged at 4000 rpm for 20 min at room temperature. Subsequently, the liquor collected from the bottom chamber was subjected to HPLC-DAD analysis, as described below. This way, the free RSV quota (RSV_out_) was determined and the Drug Loading% (DL%) and Loading Efficacy% (LE%) values were indirectly calculated knowing the total amount of RSV in the dispersion (see above: DR%) as follows:

$$\mathrm{DL}\%=\frac{{\mathrm{RSV}}_{\mathrm{tot}}-{\mathrm{RSV}}_{\mathrm{out}}\;\left(\mathrm{mg}\right)}{\mathrm{MIX}-F\;\left(\mathrm{mg}\right)}\times 100$$

$$\mathrm{LE}\%=\frac{{\mathrm{RSV}}_{\mathrm{tot}}-{\mathrm{RSV}}_{\mathrm{out}}\;\left(\mathrm{mg}\right)}{{\mathrm{RSV}}_{\mathrm{tot}}}\times 100$$

The analyses were performed in triplicate on each prepared batch of LNPs (n = 9), and results are reported as means ± SE.

### 3.8. Dynamic Light Scattering (DLS) and Z-Potential Measurements

DLS and Z-potential measurements were performed at 25.0 ± 0.5 °C by using a Zetasizer NanoZS (Malvern Panalytical Ltd., Malvern, United Kingdom) instrument equipped with a 532 nm laser at a fixed scattering angle of 173° [71,72]. Freshly prepared LNPs samples were diluted 100-fold with MilliQ (Carlo Erba, Milan, Italy) water prior to analysis. Blank measurement of the aqueous phase containing Pluronic-F127 in the absence of LNPs were performed as a control. The intensity average hydrodynamic diameter and polydispersity index (PDI) were obtained by cumulants analysis of the correlation function. Each analysis was performed in triplicate on each prepared batch of LNPs (n = 9), and results are reported as means ± SE.

### 3.9. Scanning Electron Microscopy (SEM) Analysis

The morphology of LNP-F3 was investigated using an FEI Quanta 200 FEG SEM (FEI Company, Hillsboro, OR, USA) operating with an accelerating electron voltage of 20 keV. Electron micrographs were acquired in a high-vacuum condition (3.8 × 10^−5^ mbar) and magnified up to 300,000×, setting the horizontal field and work distance at 853 nm and 10.7 mm, respectively. The surface electrical conductivity of the LNP-F3 was obtained by placing a few drops of fresh dispersion (diluted 1:1 with MilliQ water) onto an aluminium stub and placing it into a CaCl_2_ desiccator at 4 °C for 24 h. Afterward, the dry sample was covered with a thin Au film deposited by sputtering before any investigation.

### 3.10. Evaluation of the Antioxidant Power by DPPH Assay

An amount of 2 mL of DPPH stock solution (40 µg/mL in methanol) was inserted into a quartz cuvette. Subsequently, 100 µL of freshly prepared LNP-BL or LNP-F3 dispersions as well as RSV solution in methanol (RSV concentration corresponding to the total amount of RSV inserted by the LNP-F3 sample according to the DR% value) were added, well mixed and immediately subjected to UV-Vis measurements using a Shimadzu 1700 instrument (Kyoto, Japan). The antioxidant power is expressed as residual DPPH percentage amount as a function of time (means ± SE) by monitoring each sample every 5 min for 1 h at room temperature. To accurately quantify the DPPH radical, six standard methanol solutions were prepared and analysed to construct the calibration curve as follows: λ_max_ = 515 nm; linearity range: 4–40 µg/mL, regression equation: Abs = 0.018 + 28.59 × [mg/mL], (R = 0.999). Each experiment was performed in triplicate (n = 3).

### 3.11. Evaluation of Cryoprotectants Role on LNPs Freeze Drying

Based on the obtained results, only LNP-F3 composition was further studied. Freeze drying was conducted in the absence and in the presence of four different cryoprotectants: trehalose, sorbitol, mannitol and sucrose. Three solutions for each cryoprotectant were prepared at different concentrations in citrate buffer pH 5.5 and then diluted with the freshly prepared LNP-F3 dispersion to achieve 1:3, 1:8 and 1:13 weight ratios between LNPs and cryoprotectant (Table 12).

An amount of 1.5 mL of the obtained dispersions was frozen at −80 °C (Thermo Forma ultra-freezer −86 °C mod. 902 Thermo Scientific, Waltham, MA, USA) for 24 h and then subjected to freeze drying (Labconco FreeZone^®^ 2.5 Liter Freeze Dry System, Kansas City, MO, USA) for 72 h at 0.014 mBar and −50 °C. The dried samples were re-hydrated with 0.75 mL of distilled water and redispersed with the aid of an ultrasonic bath or vortex instrument (LLG-uni TEXER 1, Lab Logistics Group, Meckenheim, Germany). Accordingly, a control sample of LNP-F3 without cryoprotectant (LNP-NC) was prepared. All samples were analysed both visually (rate and simplicity of the re-dispersion process; final aspect of the resulting dispersion) and by DLS and Z-potential measured after proper dilution, as described above. Each experiment was performed in triplicate (n = 3), and results are expressed as means ± SE. 

### 3.12. In Vitro RSV Release Studies and Kinetic Evaluation

First, the best freeze-dried sample was analysed in terms of DR%, DL% and LE% after re-dispersion. Each parameter was evaluated twice for all the previously prepared samples (n = 6). The release studies were carried out on three different samples: (i) freshly prepared LNP-F3 dispersion, indicated as LNP; (ii) freshly prepared LNP-F3 dispersion in the presence of sucrose (LNP:sucrose = 1:8; *w*/*w*), indicated as LNP-Su; (iii) the latter sample subjected to the freeze-drying process and re-dispersed in MilliQ water, indicated as LNP-Su-R.

#### 3.12.1. RSV Release Studies from LNPs to Hydrophobic Acceptor Fluids

The RSV release studies to lipophilic media were conducted using a modified Transwell (Corning Incorporated, Corning, NY, USA) support (cut off: 0.45 µm). An amount of 200 µL of each sample (RSV concentration: 0.38 mg/mL) was loaded into the Transwell support and dipped inside a beaker pre-filled with 10 mL of 1-octanol or isopropyl myristate. The whole system was kept under magnetic stirring (200 rpm), at room temperature, in the dark for 48 h. At scheduled time intervals, aliquots (1 mL) were withdrawn from the acceptor fluid, immediately replaced with the same volume of fresh acceptor fluid to maintain the sink conditions and subjected to UV-Vis measurements after methanol dilution, as described below. Furthermore, at the end of the experiment the donor fluid was collected, 100-fold diluted with methanol and subjected to HPLC-DAD analysis, as described below, to verify that the sum of the residual amount of RSV and the released one matched the total amount of RSV initially loaded into the Transwell insert. The experiments were performed in triplicate and the results, presented as released dose fraction as a function of incubation time, are reported as means ± SE (n = 3).

#### 3.12.2. RSV Release Studies from LNPs to Hydrophilic Acceptor Fluids

The RSV release studies in aqueous media were carried out using the dialysis method. Samples were diluted 10-fold with citrate buffer pH 5.5 (final RSV concentration: 0.038 mg/mL), and 1 mL of the resulting dispersions was inserted into a dialysis tube (SpectraPor^®^ Standard Grade Regenerated Cellulose, 3.5 kDa, Thermo Fisher Scientific) and further immersed in 10 mL of citrate buffer pH 5.5 or citrate buffer pH 5.5 in the presence of 0.1% (*w*/*v*) β-CD. The whole system was kept under constant stirring (350 rpm) by an orbital shaker (Thermo-Shaker PST 60HL, Biosan, Riga, Latvia) at 37 ± 1 °C, in the dark for 48 h (citrate buffer with β-CD) or 24 days (citrate buffer). At scheduled time intervals, aliquots (1 mL) were withdrawn from the acceptor fluid, immediately replaced with the same volume of fresh acceptor fluid to maintain the sink conditions and subjected to UV-Vis or HPLC-DAD measurements, as described below. Furthermore, at the end of experiment, the dispersion inside the dialysis tube was collected, 10-fold diluted with methanol and subjected to HPLC-DAD analysis to quantify the residual RSV amount into the donor compartment. The experiments were performed in triplicate and results, presented as released dose fraction as a function of incubation time, are reported as means ± SE (n = 3).

#### 3.12.3. Kinetic Evaluations

The collected release data were curve fitted with several mathematical equations usually employed to describe the release mechanism from various drug delivery systems: Zero Order, First Order, Higuchi, Hixson–Crowell, Baker–Lonsdale and Korsmeyer–Peppas. Data were elaborated by Origin Pro 8.5 (Origin Lab, Northampton, MA, USA) and Microsoft Excel (Microsoft Corporation, Redmond, WA, USA) equipped with DD Solver extension [73]. To evaluate the best fitting, the R^2^ values together with the best graphical adherence to the obtained curves were considered.

### 3.13. Cell Viability Assay

Cell viability assay was performed using BALB/3-T3 cells. Both cells and reagents were obtained from Merck (Milan, Italy), with the highest purity grade commercially available. BALB/3-T3 cells were grown in DMEM supplemented with L-glutamine (2 mM), 10% foetal bovine serum (FBS), penicillin (100 U/mL) and streptomycin (100 µg/mL). Cells were maintained in their logarithm phase by seeding them twice a week at a density of 3 × 10^5^ cells/mL in a humidified 5% CO_2_ atmosphere at 37 °C (Thermo Fisher Scientific, Waltham, MA, USA, Mod: 3543). The experiments were carried out using the freshly prepared LNP-F3 dispersion, free RSV (previously dissolved in DMSO) and the control aqueous medium used for the preparation of the LNPs (citrate buffer pH 5.5 containing Pluronic F-127, indicated as Co F127). Cells at the passage that did not exceed the number 20 were seeded in a 96-well plate (Corning Incorporated, Corning, NY, USA) at a density of 1.8 × 10^4^ cells/well, incubated overnight and then treated in the absence (control) or the presence of samples corresponding to various RSV concentrations (from 2.5 to 50 μM) for 24 h. The Co F127 sample was evaluated at the same dilution of the LNP-F3 samples needed to achieve 2.5, 10 and 50 μM of RSV. After incubation, the medium was carefully removed and cells were washed with 200 μL of PBS pH 7.4, and then 200 μL of fresh medium containing MTT (5 mg/mL) was added. Plates were incubated for an additional 2 h at 37 °C and then centrifuged at 1000 rpm for 5 min (Allegra X12, Beckman Coulter, Life Sciences, Milan, Italy). The supernatant was discarded and replaced with 100 µL of DMSO to dissolve the previously precipitated formazan crystals. The absorbance at 570 nm was measured with a LTEK A-302 plate reader (INNO, Seongnam, Republic of Korea) and employed to calculate the cell viability %, referring to the absorbance of untreated control cells (100% of viability). Each experiment was performed three times in triplicate (n = 9), and the results are expressed as means ± standard deviation (SD).

### 3.14. Scratch Wound Healing Assay

The IMR-90 (ATCC CCL-186™) cell line of fibroblasts isolated from normal lung tissue derived from a 16-week-old female was cultured in DMEM medium supplemented with glutamine, 10% heat-inactivated foetal bovine serum (FBS), 100 U/mL penicillin and 100 mg/L streptomycin, as previously reported [74,75]. Cultures were maintained in a humidified atmosphere containing 5% CO_2_ at 37 °C. To evaluate cell migration, cells were plated in a 24-well plate and grown to confluency. A sterile 200 µL pipette tip was used to create a scratch in the cell layer. Following wound generation, cells were then treated with 5, 10 or 20 µM of free RSV (previously dissolved in DMSO) and LNP-F3/LNP-BL dispersion properly diluted in culture media to achieve the same active concentrations. Images were captured under a phase contrast microscope at ×40 magnification (Olympus IX70; Olympus Corporation, Tokyo, Japan) immediately or after 6, 21 and 48 h of wound generation, to evaluate wound closure. Analogously, the experiments were repeated by culturing the scratched cells in the absence of FBS and collecting wound images at 6 h. The wound area at the scheduled time points was measured using ImageJ software. The percentage of wound closure was calculated as wound area at a given time compared to the initial wound surface. The experiments were performed in triplicate and images were recorded in duplicate on each single well (n = 6). Results are expressed as means ± standard deviation (SD).

### 3.15. Inhibition of Biofilm Formation

*S. aureus* (reference strain: ATCC 12973) was cultivated on Tryptic Soy Agar, according to the previously reported protocol [76]. The bacteria, 0.5 McFarland (1.5 × 10^8^ cells), were added in a 96-well plate, together with samples containing increasing RSV concentration (from 2 to 32 µg/mL) and incubated at 37 °C for 24 h. Afterwards, the planktonic cells were removed by washing with 0.85% phosphate saline buffer (PBS), while the adherent Biofilm was fixed by adding 250 µL of absolute methanol and incubating for 15 min. The so-fixed biofilm was stained with 250 µL of 0.5% crystal violet solution for 15 min and resolubilized in 150 µL of 33% acetic acid for 15 min. The optical density (OD) of the resulting solution was measured at 570 nm using a microtiter plate reader (Multiskan Go, Thermo Fisher Scientific, Waltham, MA, USA). The selected strain was classified as a strong biofilm producer based on the cut-off OD (ODc). The latter was established by evaluating three standard deviations above the mean OD of the untreated control. The strain was then classified as follows: OD ≤ ODc = no biofilm producer, ODc < OD ≤ (2 × ODc) = weak biofilm producer, 2 ODc < OD ≤ (4 × ODc) = moderate biofilm producer, (4 × ODc) < OD = strong biofilm producer [77]. The experiments were carried out in triplicate (n = 3) using free RSV (previously dissolved in DMSO), the freshly prepared LNP-F3 dispersion (diluted to achieve the same RSV concentrations evaluated for the free active) and LNP-BL (diluted according to the LNP-F3 sample). Results (means ± SE) are reported as biofilm formation %, referring to the OD of untreated control cells (100% of biofilm formation).

### 3.16. RSV Quantitative Analysis

#### 3.16.1. UV-Vis Analysis

The UV-Vis analyses were performed using a Shimadzu 1601 instrument (Kyoto, Japan). The linearity of the analytical procedure was statistically determined by regression analysis of five or more standard concentrations in the range below, specified and evaluated in triplicate. The accuracy and precision of the method were evaluated by analysing three samples of the drug with defined concentrations. Two calibration curves were constructed:

- Methanol → λ_max_ = 305 nm; linearity range: 0.0001–0.0050 mg/mL; linear regression: Abs = 0.016 + 149.74 × C_RSV_ [mg/mL] (R = 0.999), used to calculate the DR%, and for RSV quantification into the lipid mixture and during the in vitro release studies to hydrophobic media.
- Citrate buffer pH 5.5 in the presence of 0.1% (*w*/*v*) of β-CD → λ_max_ = 305 nm; linearity range: 0.0001–0.0075 mg/mL; linear regression: Abs = 0.014 + 114.70 × C_RSV_ [mg/mL], (R = 0.999), used to quantify RSV during the in vitro release studies to hydrophilic media.

No interferences between RSV and the other components of the formulations (lipids, surfactants, salt, etc.) were observed at the testing concentrations, and no changes in terms of λ_max_ were noted. Intra-day and inter-day variations were lower than instrument sensibility.

#### 3.16.2. HPLC-DAD Analysis

HPLC-DAD analyses were performed using an Agilent Instrument 1260 Infinity (Agilent Technologies, Santa Clara, CA, USA) equipped with a Quaternary Pump G1311B, a Diode Array Detector 1260 Infinity II, an autosampler, a column oven and a computer integrating apparatus (OpenLAB ChemStation 3D UV Workstation, Santa Clara, CA, USA). Chromatographic separation was achieved by a reversed-phase column Ace^®^ Excel Super C18 (5U, 100A, size 125 × 4.60 mm; VWR International, Radnor, PA, USA) thermostated at 25 ± 1 °C by injecting 20 µL of each sample and employing a 0.1% (*v*/*v*) TFA solution in MilliQ water (solvent A) and acetonitrile (solvent B), according to the following time program: 0–2 min A:B = 70:30; 2–8 min A:B = 20:80; 8–12 min A:B = 70:30. The flow rate was set at 1 mL/min, and the DAD integration lambda at 305 nm (DAD range: 190–800 nm). In these conditions, RSV retention time was 5.3 min. The calibration curve was constructed by injecting six RSV standard solutions into methanol:

- Linearity range: 0.00005–0.00010 mg/mL; linear regression: Area = 1.062 + 152,464.17 × C_RSV_ [mg/mL], (R = 0.999).

Intra-day and inter-day variations were lower than instrument sensibility. HPLC-DAD studies were conducted to calculate the DL% and LE% values and to quantify RSV, both during the in vitro release studies to hydrophilic media and as residual amount into the donor chamber at the end of all the in vitro release studies.

### 3.17. Data Analysis

Data are expressed as mean ± standard error (SE) or standard deviation (SD). All differences were statistically evaluated by one-way analysis of variance (T-test), with the minimum level of significance set at *p* < 0.05.

## 4. Conclusions

Rapid and efficient wound healing is fundamental to restoring the functionality of injured tissue. In this context, the use of natural compounds with well-known antioxidant and anti-inflammatory activities could act as a co-adjuvant to the natural regeneration processes, thus representing a smart strategy characterized by high efficacy and low side effects. Additionally, the use of multicomponent lipid nanoparticles constituted by functional ingredients could further enhance the effectiveness of the proposed formulation as a whole, acting as an adjuvant to the active’s properties. 

In this work, RSV-loaded multicomponent LNPs were accurately designed as a functional drug delivery platform for several purposes, including wound healing. 

Firstly, each component of the nanoparticles was carefully selected to achieve a homogeneous and reproducible starting lipid mixture characterized by a suitable melting temperature range and loaded RSV in amorphous form. To confer additional properties, such as antibacterial and permeation enhancer ones, Glycyrrhetinic acid and Menthol were added to the mixture. By a top-down high-energy method (homogenization followed by high-frequency sonication), optimized solid lipid nanoparticles (LNP-F3) with a proper particle size (162.86 ± 3.12), PDI (0.267 ± 0.010), Z-potential (−21.40 ± 7.33) values and high RSV encapsulation efficiency (LE% = 95.17 ± 0.25%) were obtained. Furthermore, sucrose was proven to be an excellent cryoprotectant to store LNPs by freeze drying. Indeed, according to the in vitro release studies, the presence of sucrose in the reconstituted dispersion as well as the freeze-drying process did not affect the RSV discharge behaviour from LNPs. In particular, the RSV-loaded LNPs were confirmed as a suitable drug delivery platform able to release RSV to both hydrophilic acidic media (simulating the wound inflammatory exudate) as well as to hydrophobic fluids (simulating the target biological tissue). By curve fitting with several kinetic equations, from the release data it was observed that RSV accumulation into the lipophilic environment is governed by a Fickian diffusion process (First Order equation), while the RSV discharge mechanism to hydrophilic acidic fluids is well described by the Korsmeyer–Peppas model, resulting in unconventional *n* values (*n* < 0.5), probably related to the hydrophobic nature of the LNPs, which create a barrier to water entry and thus a RSV prolonged release. Last but not least, the biological evaluations carried out against fibroblasts demonstrated LNPs cytocompatibility and highlighted their wound healing properties due to enhanced cell migration. Particularly, the proposed LNPs seem to be able to interact with cells, thus significantly magnify RSV biological actions. This led to greatly proliferative and wound healing effects, even at extremely low RSV doses (5 μM of RSV corresponding to LNPs 22 μg/mL). At the same low doses, the proposed LNPs also displayed interesting antibiofilm properties against *S. aureus*. Based on the obtained results, the here proposed RSV-loaded LNPs were confirmed as a promising delivery platform for RSV administration, leading to overcoming all of its intrinsic limitations and demonstrating the platform as being a potentially useful tool to promote injured tissue regeneration, both directly (wound closure) and indirectly (scavenging and antibiofilm actions).

## Figures and Tables

**Table 1:** Melting temperature of MIX-F and its components.

Sample	Melting Point (°C)
LB	Liquid at room temperature
GMS	58–62
GA	296
Menthol	42.4
RSV	257
MIX-F	57–61

**Table 2:** Particle size (nm), PDI and Z-potential (mV) of the LNP dispersions reported as means ± SE (n = 9).

Sample	Intensity (nm)	PDI	Z-Potential (mV)
LNP-F1	101.17 ± 6.68	0.279 ± 0.001	−22.0. ± 10.10
LNP-F2	126.10 ± 8.30	0.337 ± 0.033	−21.50 ± 9.16
LNP-F3	162.86 ± 3.12	0.267 ± 0.010	−21.40 ± 7.33

**Table 3:** Quantitative evaluation of RSV as DR%, DL% and LE%. Means ± SE (n = 9).

Formula Code	DR%	DL%	LE%
LNP-F1	99.12 ± 1.65	4.48 ± 0.01	99.21 ± 0.14
LNP-F2	95.69 ± 0.35	4.72 ± 0.01	99.01 ± 0.22
LNP-F3	96.82 ± 1.34	4.20 ± 0.01	95.17 ± 0.25

**Table 4:** Average particle size (nm), PDI and Z-potential (mV) of the freshly prepared LNP-F3 and the same sample subjected to freeze drying in the absence (LNP-NC) or presence of the cryoprotectants at three different weight ratios. Means ± SE (n = 6).

Samples	Cryo	Ratio LNP:Cryo (*w*/*w*)	Intensity (nm)	PDI	Z-Potential (mV)
LNP-F3	Not Applicable	-	162.86 ± 3.12	0.267 ± 0.010	−21.40 ± 7.33
LNP-NC	-	-	624.65 ± 54.75	0.541 ± 0.139	−0.55 ± 5.13
LNP-T (A)	Trehalose	1:3	640.25 ± 124.35	0.516 ± 0.055	−0.21 ± 7.93
LNP-T (B)		1:8	519.30 ± 222.10	0.644 ± 0.059	0.07 ± 7.99
LNP-T (C)		1:13	577.10 ± 7.70	0.625 ± 0.063	0.52 ± 5.47
LNP-So (A)	Sorbitol	1:3	372.55 ± 72.85	0.479 ± 0.029	−0.79 ± 4.96
LNP-So (B)		1:8	566.05 ± 108.55	0.553 ± 0.026	−0.75 ± 3.61
LNP-So (C)		1:13	588.20 ± 32.70	0.549 ± 0.046	−0.43 ± 5.76
LNP-M (A)	Mannitol	1:3	1442.50 ± 502.50	0.888 ± 0.112	−0.80 ± 5.04
LNP-M (B)		1:8	603.90 ± 2.00	0.662 ± 0.014	−0.70 ± 5.89
LNP-M (C)		1:13	759.00 ± 258.00	0.698 ± 0.106	−0.23 ± 6.12
LNP-Su (A)	Sucrose	1:3	403.40 ± 12.30	0.513 ± 0.055	−1.59 ± 8.22
LNP-Su (B)		1:8	340.35 ± 57.35	0.247 ± 0.078	−1.50 ± 5.80
LNP-Su (C)		1:13	331.40 ± 43.00	0.507 ± 0.025	−1.35 ± 4.40

**Table 5:** RSV quantitative analysis reported in terms of DR%, DL% and LE% ± SE (n = 6) for the freshly prepared LNP-F3 and re-dispersed LNP-Su (B) sample after freeze drying.

Samples	DR%	DL%	LE%
LNP-F3	96.82 ± 1.34	4.20 ± 0.01	95.17 ± 0.25
LNP-Su (B)	95.81 ± 1.61	4.22 ± 0.02	96.57 ± 0.25

**Table 6:** Kinetic equations employed to determine the main mechanism governing the release of RSV from the proposed LNPs to both hydrophilic and hydrophobic environments.

Code	Models	Equations	Description of the Model
**1**	Zero Order	$\frac{M_{t}}{M_{\infty }}=k_{0}\;\cdot t\;\;$	This describes a constant drug release as a function of time [55]. It was chosen to evaluate where a pseudo-zero order condition occurred in the initial release stage (until 6−7 h). Fitting results are reported in the Supplementary Material in Tables S1–S3
**1a**	Zero Order with T_lag_	$\frac{M_{t}}{M_{\infty }}=k_{0}\;\cdot \left(t-T_{lag}\right)$	
**2**	First Order	$\frac{M_{t}}{M_{\infty }}=1\;\cdot \left(1-e^{-k_{1}\cdot t}\right)$	This describes a Fickian diffusion process that depends on the residual drug concentration as a function of time [56]. It mathematically tends to a plateau, which could be the complete release (dose fraction: 1) or an F_MAX_. Fitting results are reported in Table 10 and in the Supplementary Material in Tables S4 and S5
**2a**	First Order with T_lag_	$\frac{M_{t}}{M_{\infty }}=1\;\cdot \left[1-e^{-k_{1}\cdot \left(t-T_{lag}\right)}\;\right]$	
**2b**	First Order with F_max_	$\frac{M_{t}}{M_{\infty }}=F_{max}\;\cdot \left(1-e^{-k_{1}\cdot t}\right)$	
**2c**	First Order with F_max_ and T_lag_	$\frac{M_{t}}{M_{\infty }}=F_{max}\;\cdot \left[1-e^{-k_{1}\cdot \left(t-T_{lag}\right)}\;\right]$	
**3**	Higuchi	$\frac{M_{t}}{M_{\infty }}=k_{H}\;\cdot \;t^{0.5}$	This investigates drug diffusion through an inert matrix (e.g., lipid nanoparticles in aqueous media) [57]. Fitting results are reported in the Supplementary Material in Tables S6–S8
**3a**	Higuchi with T_lag_	$\frac{M_{t}}{M_{\infty }}=k_{H}\;\cdot {\left(t-T_{lag}\right)}^{0.5\;}$	
**4**	Baker–Lonsdale	$\frac{3}{2}\cdot \left[1-{\left(1-\frac{M_{t}}{M_{\infty }}\right)}^{\frac{2}{3}}\right]-\frac{M_{t}}{M_{\infty }}=k_{BL}\cdot t$	This is derived from the Higuchi model but considers the monolithic drug delivery device as a sphere (e.g., micro- and nano-spheres) [58]. Fitting results are reported in the Supplementary Material in Tables S9–S11
**4a**	Baker–Lonsdale with T_lag_	$\frac{3}{2}\cdot \left[1-{\left(1-\frac{M_{t}}{M_{\infty }}\right)}^{\frac{2}{3}}\right]-\frac{M_{t}}{M_{\infty }}=k_{BL}\cdot \left(t-T_{lag}\right)$	
**5**	Hixson–Crowell	$\frac{M_{t}}{M_{\infty }}=1\cdot \left[1-{\left(1-k_{hc}\cdot t\right)}^{3}\right]$	This expresses the rate of dissolution based on the cube root of the particles and describes the release from systems characterized by a change in terms of surface area and diameter (e.g., particles or tablets) [59]. Fitting results are reported in the Supplementary Material in Tables S12–S14
**5a**	Hixson–Crowell with T_lag_	$\frac{M_{t}}{M_{\infty }}=1\cdot \left\{1-{\left[1-k_{hc}\cdot (t-T_{lag}\right]}^{3}\right\}$	
**6**	Korsmeyer–Peppas	$\frac{M_{t}}{M_{\infty }}=k_{KP}\cdot t^{n}$	This is a semi-empirical model used when the drug release mechanism is unknow or more than one type of phenomena is involved. The exponent value “*n*” gives information regarding the main release process: *n* = 0.5, Fickian diffusion (Case I); 0.5 < *n* < 1, non-Fickian or anomalous transport governed by both diffusion and swelling; *n* = 1, non-Fickian model governed by swelling and/or relaxation (Case II); *n* > 1, non-Fickian model governed by tension and breaking of the vehicle structure (Super Case II) [60]. Fitting results are reported in Tables 8 and 9 and in the Supplementary Material in Table S15
**6a**	Korsmeyer–Peppas with T_lag_	$\frac{M_{t}}{M_{\infty }}=k_{KP}\cdot {\left(t-T_{lag}\right)}^{n}$	

k_0_, k_1_, k_H_, k_BL_, k_hc_ and k_KP_ are the constant parameters for Zero Order, First Order, Higuchi, Baker–Lonsdale, Hixson–Crowell and Korsmeyer–Peppas, respectively. $\frac{M_{t}}{M_{\infty }}$ is the dose fraction of RSV released as a function of time.

**Table 7:** Curve-fitting parameters calculated when considering the First Order model for RSV release studies to 1-octanol. Mean ± SE (n = 3).

Kinetic Model	Time	LNP	LNP-Su	LNP-Su-R
**2**	**28 h**	k_1_= 7.2·10^−4^ ± 7·10^−5^ R^2^ = 0.9975	k_1_ = 4.810^−4^ ± 2·10^−5^ R^2^ = 0.99500	k_1_= 9.8·10^−4^ ± 2·10^−4^ R^2^ = 0.94620
	**48 h**	k_1_ = 7.0·10^−4^ ± 7·10^−5^ R^2^ = 0.98890	k_1_= 4.6·10^−4^ ± 2·10^−5^ R^2^ = 0.98460	k_1_ = 9.6·10^−4^ ± 2.1·10^−4^ R^2^ = 0.92220
**2a**	**28 h**	k_1_ = 7.4·10^−4^ ± 6·10^−5^ t_lag_ = 0.186 ± 0.001 R^2^ = 0.99780	k_1_ = 5.0·10^−4^ ± 2·10^−5^ t_lag_ = 0.450 ± 0.002 R^2^ = 0.99690	k_1_ = 7.8·10^−4^ ± 1.2·10^−4^ t_lag_ = −1.159 ± 0.001 R^2^ = 0.96110
	**48 h**	k_1_ = 7.1·10^−4^ ± 6·10^−5^ t_lag_ = 0.052 ± 0.054 R^2^ = 0.98890	k_1_ = 4.7·10^−4^ ± 2·10^−5^ t_lag_ = 0.205 ± 0.204 R^2^ = 0.98490	k_1_ = 7.1·10^−4^ ± 1.1·10^−5^ t_lag_ = −1.568 ± 0.099 R^2^ = 0.94150
**2b**	**28 h**	k_1_ = 0.093 ± 0.005 F_MAX_ = 0.870 ± 0.016 R^2^ = 0.98746	k_1_ = 0.082 ± 0.008 F_MAX_ = 0.793 ± 0.008 R^2^ = 0.98677	k_1_ = 0.146 ± 0.001 F_MAX_ = 0.833 ± 0.025 R^2^ = 0.99187
	**48 h**	k_1_ = 0.093 ± 0.005 F_MAX_ = 0.870 ± 0.016 R^2^ = 0.99146	k_1_ = 0.081 ± 0.007 F_MAX_ = 0.793 ± 0.008 R^2^ = 0.98705	k_1_ = 0.146 ± 0.013 F_MAX_ = 0.833 ± 0.025 R^2^ = 0.99195
**2c**	**28 h**	k_1_ = 0.109 ± 0.006 F_MAX_ = 0.870 ± 0.016 t_lag_ = 0.687 ± 0.158 R^2^ = 0.99548	k_1_ = 0.092 ± 0.001 F_MAX_ = 0.793 ± 0.008 t_lag_ = 0.936 ± 0.032 R^2^ = 0.99984	k_1_ = 0.177 ± 0.010 F_MAX_ = 0.833 ± 0.025 t_lag_ = 0.601 ± 0.083 R^2^ = 0.99855
	**48 h**	k_1_ = 0.109 ± 0.006 F_MAX_ = 0.870 ± 0.016 t_lag_ = 0.690 ± 0.150 R^2^ = 0.99698	k_1_ = 0.092 ± 0.001 F_MAX_ = 0.793 ± 0.008 t_lag_ = 0.936 ± 0.032 R^2^ = 0.99983	k_1_ = 0.177 ± 0.009 F_MAX_ = 0.833 ± 0.025 t_lag_ = 0.601 ± 0.078 R^2^ = 0.99859

**Table 8:** Curve-fitting parameters calculated when considering the Korsmeyer–Peppas model for the RSV release studies to citrate buffer pH 5.5. Mean ± SE (n = 3).

Kinetic Model	Time	LNP	LNP-Su	LNP-Su-R
**6**	**24 days**	k_KP_ = 0.490 ± 0.01 *n* = 0.222 ± 0.007 R^2^ = 0.98720	k_KP_ = 0.368 ± 0.020 *n* = 0.313 ± 0.018 R^2^ = 0.98435	k_KP_ = 0.315 ± 0.011 *n* = 0.354 ± 0.012 R^2^ = 0.99618
**6a**	**24 days**	k_KP_ = 0.513 ± 0.007 t_lag_ = 0.037 ± 0.004 *n* = 0.205 ± 0.001 R^2^ = 0.99469	k_KP_ = 0.453 ± 0.012 t_lag_ = 0.158 ± 0.015 *n* = 0.245 ± 0.009 R^2^ = 0.99734	k_KP_ = 0.333 ± 0.014 t_lag_ = 0.014 ± 0.004 *n* = 0.335 ± 0.001 R^2^ = 0.99656

**Table 9:** Curve-fitting parameters calculated when considering the Korsmeyer–Peppas model for the RSV release studies to citrate buffer pH 5.5 containing 0.1% (*w*/*v*) of β-CD. Mean ± SE (n = 3).

Kinetic Model	Time	LNP	LNP-Su	LNP-Su-R
**6**	**26 h**	k_KP_ = 0.372 ± 0.007 *n* = 0.259 ± 0.009 R^2^ = 0.98834	k_KP_ = 0.264 ± 0.013 *n* = 0.366 ± 0.016 R^2^ = 0.98933	k_KP_ = 0.347 ± 0.021 *n* = 0.321 ± 0.037 R^2^ = 0.87204
	**48 h**	k_KP_ = 0.388 ± 0.007 *n* = 0.235 ± 0.005 R^2^ = 0.99249	k_KP_ = 0.315 ± 0.026 *n* = 0.290 ± 0.023 R^2^ = 0.96738	k_KP_ = 0.374 ± 0.023 *n* = 0.243 ± 0.020 R^2^ = 0.93502
**6a**	**26 h**	k_KP_ = 0.395 ± 0.017 t_lag_ = 0.520 ± 0.293 *n* = 0.240 ± 0.016 R^2^ = 0.97566	k_KP_ = 0.344 ± 0.070 t_lag_ = 1.512 ± 0.863 *n* = 0.288 ± 0.064 R^2^ = 0.92034	k_KP_ = 0.528 ± 0.070 t_lag_ = 1.764 ± 0.342 *n* = 0.163 ± 0.068 R^2^ = 0.78983
	**48 h**	k_KP_ = 0.437 ± 0.084 t_lag_ = 1.437 ± 2.279 *n* = 0.205 ± 0.051 R^2^ = 0.91111	k_KP_ = 0.539 ± 0.272 t_lag_ = 5.427 ± 4.294 *n* = 0.153 ± 0.135 R^2^ = 0.62946	k_KP_ = 0.440 ± 0.022 t_lag_ = 0.942 ± 0.110 *n* = 0.201 ± 0.016 R^2^ = 0.95874

**Table 10:** Composition of 1 g of each lipid mixtures reported as mg of each component.

Formula Code	LB	GMS	GA	M	RSV
MIX-A	230	690	30	-	50
MIX-B	365	530	30	-	75
MIX-C	350	520	30	-	100
MIX-D	400	470	30	-	100
MIX-E	320	600	30	-	50
MIX-F	300	600	30	20	50
MIX-BL	300	600	30	20	-

**Table 11:** Composition of the prepared LNP dispersions.

Formula Code	MIX Type	MIX Amount (mg)	Citrate Buffer pH 5.5 (mL)	Pluronic F-127 (mg)
LNP-F1	MIX-F	100	40	400
LNP-F2	MIX-F	200	40	400
LNP-F3	MIX-F	300	40	400
LNP-BL	MIX-BL	300	40	400

**Table 12:** LNP samples subjected to the freeze-drying process: cryoprotectant type and weight ratio.

Formula Code	Ratio LNP:Cryo (*w*/*w*)	Cryoprotectant
LNP-T (A)	1:3	Trehalose
LNP-T (B)	1:8	
LNP-T (C)	1:13	
LNP-So (A)	1:3	Sorbitol
LNP-So (B)	1:8	
LNP-So (C)	1:13	
LNP-M (A)	1:3	Mannitol
LNP-M (B)	1:8	
LNP-M (C)	1:13	
LNP-Su (A)	1:3	Sucrose
LNP-Su (B)	1:8	
LNP-Su (C)	1:13	
LNP-NC	-	Absent

## Data Availability

Not applicable.

## Supplementary Materials

The following supporting information can be downloaded at: https://www.mdpi.com/article/10.3390/ijms24098382/s1.
