# Supplementary material for: Multicomponent Antibiofilm Lipid Nanoparticles as Novel Platform to Ameliorate Resveratrol Properties: Preliminary Outcomes on Fibroblast Proliferation and Migration
Source: Int J Mol Sci. 2023 May 6;24(9):8382. doi: 10.3390/ijms24098382 (PMC10179555; doi:10.3390/ijms24098382)
Supplement: Supplementary file 1 [file ijms-24-08382-s001.zip › ijms-2338761 - Supplementary materials.pdf]

International Journal of *Molecular Sciences*

MDPI

Supplementary material

Article

Multicomponent Antibiofilm Lipid Nanoparticles as Novel Platform to Ameliorate Resveratrol Properties: Preliminary Outcomes on fibroblast Proliferation and Migration

Giuseppe Angellotti[1,2,x], Giulia Di Prima[2,x], Fabio D'Agostino[3], Emanuela Peri[2], Maria Rita Tricoli[4], Elena Belfiore[1,2], Mario Allegra[2], Patrizia Cancemi[2] and Viviana De Caro[2*]

[1] Department of Surgical, Oncological and Oral Sciences, University of Palermo, 90127 Palermo, Italy. giuseppe.angellotti@unipa.it (GA), elena.belfiore@unipa.it (EB)
[2] Department of Biological, Chemical and Pharmaceutical Sciences and Technologies, University of Palermo, 90123 Palermo, Italy. giulia.diprima@unipa.it (GDP), emanuela.peri@unipa.it (EP), mario.allegra@unipa.it (MA), patrizia.cancemi@unipa.it (PC), viviana.decaro@unipa.it (VDC)
[3] Institute for the Study of Anthropogenic Impacts and Sustainability in the Marine Environment, National Research Council (IAS—CNR), Campobello di Mazara, 91021 Trapani, Italy. fabio.dagostino@cnr.it (FD)
[4] Department of Health Promotion, Maternal-Childhood, Internal Medicine of Excellence G. D'Alessandro, Section of Microbiology, University of Palermo, 90127 Palermo, Italy, mariarita.tricoli@unipa.it. (MRT)
[x] The authors equally contributed to this work
* Correspondence: viviana.decaro@unipa.it; Tel.: +3909123891926

Data reported as supplementary material:

- **Table S1**: Curve-fitting parameters calculated when considering the *Zero* order model for RSV release studies to 1-octanol.
- **Table S2**: Curve-fitting parameters calculated when considering the Zero order model for the RSV release studies to citrate buffer pH 5.5.
- **Table S3**: Curve-fitting parameters calculated when considering the Zero order model for the RSV release studies to citrate buffer pH 5.5 containing 0.1% (w/v) of β-CD.
- **Table S4**: Curve-fitting parameters calculated when considering the First order model for the RSV release studies to citrate buffer pH 5.5.
- **Table S5**: Curve-fitting parameters calculated when considering the First order model for the RSV release studies to citrate buffer pH 5.5 containing 0.1% (w/v) of β-CD.
- **Table S6**: Curve-fitting parameters calculated when considering the *Higuchi* model for RSV release studies to 1-octanol.
- **Table S7**: Curve-fitting parameters calculated when considering the Higuchi model for the RSV release studies to citrate buffer pH 5.5.
- **Table S8**: Curve-fitting parameters calculated when considering the Higuchi model for the RSV release studies to citrate buffer pH 5.5 containing 0.1% (w/v) of β-CD.
- **Table S9**: Curve-fitting parameters calculated when considering the Baker-Lonsdale model for RSV release studies to 1-octanol.
- **Table S10**: Curve-fitting parameters calculated when considering the Baker-Lonsdale model for the RSV release studies to citrate buffer pH 5.5.

- **Table S11**: Curve-fitting parameters calculated when considering the Baker-Lonsdale model for the RSV release studies to citrate buffer pH 5.5 containing 0.1% (w/v) of β-CD.
- **Table S12:** Curve-fitting parameters calculated when considering the Hixon-Crowell model for RSV release studies to 1-octanol.
- **Table S13:** Curve-fitting parameters calculated when considering the Hixon-Crowell model for the RSV release studies to citrate buffer pH 5.5.
- **Table S14:** Curve-fitting parameters calculated when considering the Hixon-Crowell model for the RSV release studies to citrate buffer pH 5.5 containing 0.1% (w/v) of β-CD.
- **Table S15:** Curve-fitting parameters calculated when considering the Kormeyer-Peppas model for RSV release studies to 1-octanol.

Table S1. Curve-fitting parameters calculated when considering the Zero order model for RSV release studies to 1-octanol. Mean ± SE (n=3).

Kinetic model	Time	NLC	NLC-Su	NLC-Su-R
**1**	**6 h**	k_0 = 0.062 ± 0.005<br>R^2 = 0.98830	k_0 = 0.043 ± 0.003<br>R^2 = 0.94660	k_0 = 0.087 ± 0.010<br>R^2 = 0.95440
**1a**	**6 h**	k_0= 0.065 ± 0.001<br>t_{lag}= 0.23 ± 0.22<br>R^2 = 0.99150	k_0 = 0.053 ± 0.003<br>t_{lag}= 0.83 ± 0.13<br>R^2 = 0.99090	k_0 = 0.083 ± 0.003<br>t_{lag}= -0.21 ± 0.35<br>R^2 = 0.95740

Table S2. Curve-fitting parameters calculated when considering the Zero order model for the RSV release studies to citrate buffer pH 5.5. Mean ± SE (n=3).

Kinetic model	Time	NLC	NLC-Su	NLC-Su-R
**1**	**6 h**	k_0 = 0.066 ± 0.003<br>R^2 = 0.82946	k_0 =0.051 ± 0.003<br>R^2 = 0.94947	k_0 = 0.029 ± 0.001<br>R^2 = 0.96763
**1a**	**6 h**	k_0 = 0.052 ± 0.007<br>t_{lag} = -1.50 ± 0.85<br>R^2 = 0.89870	k_0 = 0.045 ± 0.003<br>t_{lag} = -0.39 ± 0.05<br>R^2 = 0.97025	k_0 = 0.033 ± 0.001<br>t_{lag} = 0.27 ± 0.06<br>R^2 = 0.99802

Table S3. Curve-fitting parameters calculated when considering the Zero order model for the RSV release studies to citrate buffer pH 5.5 containing 0.1% (w/v) of β-CD. Mean ± SE (n=3).

Kinetic model	Time	NLC	NLC-Su	NLC-Su-R
**1**	**7 h**	*Null*	k_0 = 0.087 ± 0.006<br>R^2 = 0.55293	*null*
**1a**	**7 h**	k_0 = 0.038 ± 0.007<br>t_{lag} = -9.59 ± 2.78	k_0 = 0.055 ± 0.003<br>t_{lag} = -3.49 ± 0.450	k_0 = 0.078 ± 0.010<br>t_{lag} = -3.25 ± 0.76

		R^2 = 0.80396	R^2 = 0.98101	R^2 = 0.90384

Table S4. Curve-fitting parameters calculated when considering the First order model for the RSV release studies to citrate buffer pH 5.5. Mean ± SE (n=3).

Kinetic model	Time	NLC	NLC-Su	NLC-Su-R
2	24 days	k_1 = 1.808 ± 0.538<br>R^2 = 0.62881	k_1 = 0.166 ± 0.005<br>R^2 = 0.99303	k_1 = 0.131 ± 0.009<br>R^2 = 0.96714
2a	24 days	k_1 = 0.155 ± 0.013<br>t_{lag} = -2.366 ± 0.338<br>R^2 = 0.96305	k_1 = 0.162 ± 0.004<br>t_{lag} = -0.247 ± 0.088<br>R^2 = 0.99478	k_1 = 0.122 ± 0.006<br>t_{lag} = -0.862 ± 0.200<br>R^2 = 0.98930

Table S5. Curve-fitting parameters calculated when considering the First order model for the RSV release studies to citrate buffer pH 5.5 containing 0.1% (w/v) of β-CD. Mean ± SE (n=3).

Kinetic model	Time	NLC	NLC-Su	NLC-Su-R
2	26 h	k_1 = 0.151 ± 0.009<br>R^2 = 0.63674	k_1 = 0.106 ± 0.012<br>R^2 = 0.87575	k_1 = 0.240 ± 0.013<br>R^2 = 0.088414
	48 h	k_1 = 0.151 ± 0.009<br>R^2 = 0.87948	k_1 = 0.101 ± 0.012<br>R^2 = 0.91686	k_1 = 0.240 ± 0.015<br>R^2 = 0.94788
2a	26 h	k_1 = 0.063 ± 0.008<br>t_{lag}= -7.767 ± 1.684<br>R^2 = 0.94834	k_1 = 0.070 ± 0.004<br>t_{lag}= -3.576 ± 0.645<br>R^2 = 0.98257	k_1 = 0.172 ± 0.019<br>t_{lag}= -0.980 ± 0.373<br>R^2 = 0.94651
	48 h	k_1 = 0.058 ± 0.006<br>t_{lag}= -8.752 ± 1.502<br>R^2 = 0.98359	k_1 = 0.064 ± 0.004<br>t_{lag}= -4.322 ± 0.742<br>R^2 = 0.98723	k_1= 0.172 ± 0.027<br>t_{lag}= -0.990 ± 0.523<br>R^2 = 0.96506

Table S6. Curve-fitting parameters calculated when considering the Higuchi model for RSV release studies to 1-octanol. Mean ± SE (n=3).

Kinetic model	Time	NLC	NLC-Su	NLC-Su-R
3	28 h	k_H = 0.156 ± 0.006<br>R^2 = 0.95030	k_H = 0.128 ± 0.004<br>R^2 = 0.90860	k_H = 0.166 ± 0.009<br>R^2 = 0.95960
	48 h	k_H = 0.146 ± 0.005<br>R^2 = 0.92930	k_H = 0.124 ± 0.003<br>R^2 = 0.92140	k_H = 0.151 ± 0.007<br>R^2 = 0.87870
	28 h	k_H = 0.171 ± 0.005<br>t_{lag}= 1.659 ± 0.134	k_H = 0.145 ± 0.001<br>t_{lag}= 2.306 ± 0.379	k_H = 0.169 ± 0.005<br>t_{lag}= 0.386 ± 0.005

		R^2 = 0.99510	R^2 = 0.99500	R^2 = 0.96310
**3a**	**48 h**	k_H = 0.153 ± 0.003<br>t_{lag}= 1.089± 0.004<br>R^2 = 0.94750	k_H = 0.134 ± 0.001<br>t_{lag}= 2.000± 0.001<br>R^2 = 0.97370	k_H = 0.148 ± 0.002<br>t_{lag}= -0.575± 0.010<br>R^2 = 0.88090

Table S7. Curve-fitting parameters calculated when considering the Higuchi model for the RSV release studies to citrate buffer pH 5.5. Mean ± SE (n=3).

Kinetic model	Time	NLC	NLC-Su	NLC-Su-R
**3**	**24 days**	k_H = 0.224 ± 0.010<br>R^2 = 0.61968	k_H = 0.210 ± 0.004<br>R^2 = 0.92706	k_H = 0.202 ± 0.002<br>R^2 = 0.98013
**3a**	**24 days**	k_H = 0.201 ± 0.001<br>t_{lag} = -3.242 ± 0.760<br>R^2 = 0.88933	k_H = 0.210 ± 0.004<br>t_{lag} = -0.029 ± 0.113<br>R^2 = 0.92394	k_H = 0.201 ± 0.002<br>t_{lag} = -0.201 ± 0.099<br>R^2 = 0.98413

Table S8. Curve-fitting parameters calculated when considering the Higuchi model for the RSV release studies to citrate buffer pH 5.5 containing 0.1% (w/v) of β-CD. Mean ± SE (n=3).

Kinetic model	Time	NLC	NLC-Su	NLC-Su-R
**3**	**26 h**	Null	k_H = 0.178 ± 0.005<br>R^2 = 0.91968	k_H = 0.257 ± 0.018<br>R^2 = 0.47482
	**48 h**	Null	k_H = 0.144 ± 0.005<br>R^2 = 0.76296	k_H = 0.160 ± 0.017<br>R^2 = 0.19041
**3a**	**26 h**	k_H = 0.147 ± 0.009<br>t_{lag}= -9.641 ± 1.978<br>R^2 = 0.90983	k_H = 0.166 ± 0.005<br>t_{lag}=-2.326 ± 0.843<br>R^2 = 0.96329	k_H = 0.199 ± 0.025<br>t_{lag}= -2.629 ± 1.430<br>R^2 = 0.70826
	**48 h**	k_H = 0.121 ± 0.005<br>t_{lag}= -17.133 ± 2.585<br>R^2 = 0.94388	k_H = 0.130 ± 0.006<br>t_{lag}= -8.784 ± 3.550<br>R^2 = 0.88910	k_H = 0.126 ± 0.011<br>t_{lag}= -10.880 ± 3.330<br>R^2 = 0.83860

Table S9. Curve-fitting parameters calculated when considering the Baker-Lonsdale model for RSV release studies to 1-octanol. Mean ± SE (n=3).

Kinetic model	Time	NLC	NLC-Su	NLC-Su-R
4	28 h	$k_{BL} = 6 \cdot 10^{-5} \pm 1 \cdot 10^{-5}$<br>$R^2 = 0.89340$	$k_{BL} = 4 \cdot 10^{-5} \pm 1 \cdot 10^{-5}$<br>$R^2 = 0.85960$	$k_{BL} = 7 \cdot 10^{-5} \pm 1 \cdot 10^{-5}$<br>$R^2 = 0.95420$
	48 h	$k_{BL} = 6 \cdot 10^{-5} \pm 1 \cdot 10^{-5}$<br>$R^2 = 0.91100$	$k_{BL} = 4 \cdot 10^{-5} \pm 2 \cdot 10^{-5}$<br>$R^2 = 0.88950$	$k_{BL} = 7 \cdot 10^{-5} \pm 2 \cdot 10^{-5}$<br>$R^2 = 0.94440$
4a	28 h	$k_{BL} = 8 \cdot 10^{-5} \pm 1 \cdot 10^{-5}$<br>$t_{lag} = 2.087 \pm 0.387$<br>$R^2 = 0.98570$	$k_{BL} = 5 \cdot 10^{-5} \pm 1 \cdot 10^{-5}$<br>$t_{lag} = 2.709 \pm 0.090$<br>$R^2 = 0.98940$	$k_{BL} = 9 \cdot 10^{-5} \pm 1 \cdot 10^{-5}$<br>$t_{lag} = 1.067 \pm 0.003$<br>$R^2 = 0.99600$
	48 h	$k_{BL} = 8 \cdot 10^{-5} \pm 1 \cdot 10^{-5}$<br>$t_{lag} = 2.040 \pm 0.343$<br>$R^2 = 0.98040$	$k_{BL} = 5 \cdot 10^{-5} \pm 1 \cdot 10^{-5}$<br>$t_{lag} = 2.652 \pm 0.111$<br>$R^2 = 0.98610$	$k_{BL} = 8 \cdot 10^{-5} \pm 2 \cdot 10^{-5}$<br>$t_{lag} = 0.908 \pm 0.055$<br>$R^2 = 0.97110$

Table S10. Curve-fitting parameters calculated when considering the Baker-Lonsdale model for the RSV release studies to citrate buffer pH 5.5. Mean ± SE (n=3).

Kinetic model	Time	NLC	NLC-Su	NLC-Su-R
4	24 days	$k_{BL} = 2.1 \cdot 10^{-4} \pm 1 \cdot 10^{-5}$<br>$R^2 = 0.85420$	$k_{BL} = 2 \cdot 10^{-4} \pm 1 \cdot 10^{-5}$<br>$R^2 = 0.98690$	$k_{BL} = 1.5 \cdot 10^{-4} \pm 2 \cdot 10^{-5}$<br>$R^2 = 0.94180$
4a	24 days	$k_{BL} = 2 \cdot 10^{-4} \pm 1 \cdot 10^{-5}$<br>$t_{lag} = -0.651 \pm 0.001$<br>$R^2 = 0.94800$	$k_{BL} = 1.9 \cdot 10^{-4} \pm 1 \cdot 10^{-5}$<br>$t_{lag} = -0.204 \pm 0.280$<br>$R^2 = 0.98700$	$k_{BL} = 1.3 \cdot 10^{-4} \pm 1 \cdot 10^{-5}$<br>$t_{lag} = -0.437 \pm 0.180$<br>$R^2 = 0.97790$

Table S11. Curve-fitting parameters calculated when considering the Baker-Lonsdale model for the RSV release studies to citrate buffer pH 5.5 containing 0.1% (w/v) of β-CD. Mean ± SE (n=3).

Kinetic model	Time	NLC	NLC-Su	NLC-Su-R
4	26 h	$k_{BL} = 1.3\cdot10^{-4} \pm 1\cdot10^{-5}$<br>$R^2 = 0.92450$	$k_{BL} = 1\cdot10^{-4} \pm 1\cdot10^{-5}$<br>$R^2 = 0.99510$	$k_{BL} = 1.7\cdot10^{-4} \pm 2\cdot10^{-5}$<br>$R^2 = 0.92140$
	48 h	$k_{BL} = 1\cdot10^{-4} \pm 1\cdot10^{-5}$<br>$R^2 = 0.90720$	$k_{BL} = 1\cdot10^{-4} \pm 1\cdot10^{-5}$<br>$R^2 = 0.99420$	$k_{BL} = 1\cdot10^{-4} \pm 2\cdot10^{-5}$<br>$R^2 = 0.79390$
4a	26 h	$k_{BL} = 1.1\cdot10^{-4} \pm 3\cdot10^{-5}$<br>$t_{lag} = -1.004 \pm 0.232$<br>$R^2 = 0.96700$	$k_{BL} = 1\cdot10^{-4} \pm 1\cdot10^{-5}$<br>$t_{lag} = -0.030 \pm 0.228$<br>$R^2 = 0.99510$	$k_{BL} = 1.9\cdot10^{-4} \pm 1\cdot10^{-5}$<br>$t_{lag} = 0.188 \pm 0.102$<br>$R^2 = 0.92900$
	48 h	$k_{BL} = 1\cdot10^{-4} \pm 1\cdot10^{-5}$<br>$t_{lag} = -1.125 \pm 0.262$<br>$R^2 = 0.97290$	$k_{BL} = 1\cdot10^{-4} \pm 1\cdot10^{-5}$<br>$t_{lag} = -0.048 \pm 0.224$<br>$R^2 = 0.99430$	$k_{BL} = 1\cdot10^{-4} \pm 1\cdot10^{-5}$<br>$t_{lag} = -1.367 \pm 0.529$<br>$R^2 = 0.85450$

Table S12. Curve-fitting parameters calculated when considering the Hixon-Crowell model for RSV release studies to 1-octanol. Mean ± SE (n=3).

Kinetic model	Time	NLC	NLC-Su	NLC-Su-R
5	28 h	$k_{hc} = 1.9\cdot10^{-4} \pm 2\cdot10^{-5}$<br>$R^2 = 0.98520$	$k_{hc} = 1.3\cdot10^{-4} \pm 1\cdot10^{-5}$<br>$R^2 = 0.99170$	$k_{hc} = 3.0\cdot10^{-4} \pm 9\cdot10^{-5}$<br>$R^2 = 0.86820$
	48 h	$k_{hc} = 1.9\cdot10^{-4} \pm 2\cdot10^{-5}$<br>$R^2 = 0.97160$	$k_{hc} = 1.3\cdot10^{-4} \pm 1\cdot10^{-5}$<br>$R^2 = 0.96670$	$k_{hc} = 2.4\cdot10^{-4} \pm 4\cdot10^{-5}$<br>$R^2 = 0.84290$
5a	28 h	$k_{hc} = 1.8\cdot10^{-4} \pm 1\cdot10^{-5}$<br>$t_{lag} = -0.749 \pm 0.148$<br>$R^2 = 0.99020$	$k_{hc} = 1.3\cdot10^{-4} \pm 1\cdot10^{-5}$<br>$t_{lag} = -0.164 \pm 0.270$<br>$R^2 = 0.9919$	$k_{hc} = 1.7\cdot10^{-4} \pm 1\cdot10^{-5}$<br>$t_{lag} = -3.166 \pm 0.516$<br>$R^2 = 0.93600$
	48 h	$k_{hc} = 1.8\cdot10^{-4} \pm 1\cdot10^{-5}$<br>$t_{lag} = -0.839 \pm 0.077$<br>$R^2 = 0.97640$	$k_{hc} = 1.2\cdot10^{-4} \pm 1\cdot10^{-5}$<br>$t_{lag} = -0.617 \pm 0.275$<br>$R^2 = 0.96860$	$k_{hc} = 1.6\cdot10^{-4} \pm 2\cdot10^{-5}$<br>$t_{lag} = -3.485 \pm 0.003$<br>$R^2 = 0.91100$

Table S13. Curve-fitting parameters calculated when considering the Hixon-Crowell model for the RSV release studies to citrate buffer pH 5.5. Mean ± SE (n=3).

Kinetic model	Time	NLC	NLC-Su	NLC-Su-R
**5**	**24 days**	k_{hc} = 0.056 ± 0.007<br>R^2 = 0.59389	k_{hc} = 0.043 ± 0.001<br>R^2 = 0.98505	k_{hc} = 0.031 ± 0.002<br>R^2 = 0.95725
**5a**	**24 days**	k_{hc} = 0.034 ± 0.003<br>t_{lag} = 0.120 ± 0.010<br>R^2 = 0.95243	k_{hc} = 0.042 ± 0.002<br>t_{lag} = 0.0150 ± 0.007<br>R^2 = 0.98692	k_{hc} = 0.027 ± 0.001<br>t_{lag} = 0.040 ± 0.008<br>R^2 = 0.98114

Table S14. Curve-fitting parameters calculated when considering the Hixon-Crowell model for the RSV release studies to citrate buffer pH 5.5 containing 0.1% (w/v) of β-CD. Mean ± SE (n=3).

Kinetic model	Time	NLC	NLC-Su	NLC-Su-R
**5**	**26 h**	k_{hc} = 0.045 ± 0.003<br>R^2 = 0.49112	k_{hc} = 0.037 ± 0.060<br>R^2 = 0.73342	k_{hc} = 0.061 ± 0.005<br>R^2 = 0.51923
	**48 h**	k_{hc} = 0.031 ± 0.003<br>R^2 = 0.14769	k_{hc} = 0.024 ± 0.003<br>R^2 = 0.81635	k_{hc} = 0.030 ± 0.004<br>R^2 = 0.0362
**5a**	**26 h**	k_{hc} = 0.013 ± 0.002<br>t_{lag}= -16.644 ± 2.743<br>R^2 = 0.92214	k_{hc} = 0.016 ± 0.001<br>t_{lag}= -6.570 ± 1.270<br>R^2 = 0.96225	k_{hc} = 0.044 ± 0.006<br>t_{lag}= -1.702 ± 0.550<br>R^2 = 0.92352
	**48 h**	k_{hc} = 0.011 ± 0.001<br>t_{lag}= -16.779 ± 2.793<br>R^2 = 0.97062	k_{hc} = 0.013 ± 0.001<br>t_{lag}= -9.035 ± 2.082<br>R^2 = 0.95771	k_{hc} = 0.022 ± 0.006<br>t_{lag}= -5.708 ± 2.289<br>R^2 = 0.90797

Table S15. Curve-fitting parameters calculated when considering the Kormeyer-Peppas model for RSV release studies to 1-octanol. Mean ± SE (n=3).

Kinetic model	Time	NLC	NLC-Su	NLC-Su-R
**6**	**28 h**	k_{KP} = 0.099 ± 0.011	k_{KP} = 0.057 ± 0.008	k_{KP} = 0.177 ± 0.032
		n = 0.659 ± 0.025	n = 0.775 ± 0.036	n = 0.481± 0.046
		R^2 = 0.98830	R^2 = 0.98990	R^2 = 0.96090
	**48 h**	k_{KP} = 0.134 ± 0.012	k_{KP} = 0.086 ± 0.008	k_{KP} = 0.206 ± 0.031
		n = 0.529 ± 0.002	n = 0.614 ± 0.021	n = 0.404 ± 0.034
		R^2 = 0.93110	R^2 = 0.94340	R^2 = 0.91370
**6a**	**28 h**	k_{KP} = 0.134 ± 0.011	k_{KP} = 0.112 ± 0.011	k_{KP} = 0.242 ± 0.042
		t_{lag}= 0.872 ± 0.001	t_{lag}= 1.740 ± 0.001	t_{lag}= 0.980 ± 0.001
		n = 0.571 ± 0.016	n = 0.580 ± 0.026	n = 0.386 ± 0.045
		R^2 = 0.99470	R^2 = 0.99960	R^2 = 0.9932
	**48 h**	k_{KP} = 0.221 ± 0.015	k_{KP} = 0.174 ± 0.012	k_{KP} = 0.326 ± 0.043
		t_{lag}= 1.910 ± 0.001	t_{lag}= 2.500 ± 0.005	t_{lag}= 1.865 ± 0.001
		n = 0.394 ± 0.012	n = 0.425 ± 0.001	n = 0.276 ± 0.001
		R^2 = 0.96330	R^2 = 0.97850	R^2 = 0.97090
